# The role, challenges, and solutions of laboratories in disaster medicine: a systematic review

**DOI:** 10.3389/fpubh.2025.1726280

**Published:** 2026-01-13

**Authors:** Kien Trung Tran, Ky Dinh Nguyen, Tho Ngoc Nguyen, Lam The Pham, Linh Mai Nguyen, Phong Han Nguyen, Nam Hoang Tran, Chung Thi Kim Le

**Affiliations:** 1 Hanoi Medical University, Hanoi, Vietnam; 2 Tokushima University, Tokushima, Japan; 3 Institute for Preventive Medicine and Public Health, Hanoi Medical University, Hanoi, Vietnam

**Keywords:** solutions, disaster medicine, disaster planning, emergency medical services, laboratories

## Abstract

**Objectives:**

Laboratory systems play a critical role in disaster response, yet evidence remains fragmented. This systematic review aimed to describe the roles of clinical, public health, and veterinary laboratories, specifically characterizing Point-of-Care Testing (POCT) and Mobile Laboratories (ML) as flexible operational extensions of the central laboratory system across disaster phases; identify and compare laboratory-related challenges by disaster type; and synthesize documented solutions and their effectiveness.

**Methods:**

4,323 studies published between 2000 and 2025 were identified through searches in PubMed, Embase, Scopus, grey literature, and snowballing. Study screening, data extraction, and methodological quality appraisal were conducted in accordance with the Preferred Reporting Items for Systematic Reviews and Meta-Analyses (PRISMA) 2020 statement. Risk of bias was assessed using the critical appraisal checklist for qualitative research developed by the Joanna Briggs Institute (JBI).

**Results:**

Fifty-two studies were included. While clinical, public health, and veterinary laboratories form the “National Core Layer,” POCT and rapid response mobile laboratory were identified as the “Surge Capacity Layer,” functioning as flexible extended arms. Instead of random barriers, laboratory challenges were found to align along three operational axes: (1) Scarcity (infrastructure fragility and workforce shortages), predominantly in low-resource settings; (2) Complexity (data fragmentation and quality assurance variability), driven by technological heterogeneity in high-income settings; and (3) Security (regulatory barriers and cybersecurity risks), characterizing conflict and bio-risk environments. Documented solutions showed mixed effectiveness.

**Conclusion:**

Building on these insights, we propose a structured framework to guide scalable strategies that enhance laboratory system resilience for disaster preparedness and response.

**Systematic review registration:**

The protocol was prospectively registered in PROSPERO (CRD420251053068) https://www.crd.york.ac.uk/PROSPERO/view/CRD420251053068.

## Introduction

Disasters, whether natural, human-induced, or epidemic, continue to pose substantial burdens on global health systems (1). Each year, an estimated 190 million people are affected by natural and technological hazards, and over 170 million by conflict-related crises (2, 3). From 2012 to 2017, the World Health Organization (WHO) recorded over 1,200 outbreaks in 168 countries (4). In subsequent years, the COVID-19 pandemic caused over 6.5 million deaths by 2024 (5). These impacts are especially severe in low- and middle-income countries (LMICs), where disasters are frequent and health systems are under-resourced (6). In these high-pressure contexts, laboratory systems play a central role in detecting hazards, guiding clinical management, and enabling public health decision-making (7–10). The foundational architecture of disaster laboratory systems relies on a tiered network of static clinical, public health, and veterinary facilities (9, 11). To bridge the operational gap between these centralized hubs and the crisis epicenter, RRMLs and POCT are utilized as flexible operational modalities (12–14). These platforms function as the forward-deployed ‘extended arms’ of the central laboratory network, providing essential diagnostic surge capacity and ensuring service continuity even when static infrastructure is compromised (8, 15).

However, evidence on laboratory performance in disasters remains fragmented, typically limited by the type of hazard, country, or laboratory modality. More critically, there is a stark mismatch between the burden of disasters in LMICs and the availability of context-appropriate, evidence-based laboratory guidelines (6, 8–10, 13). Most existing guidance originates from high-income countries (HICs) and does not adequately reflect the operational constraints of LMICs, where power instability, workforce shortages, and logistical barriers are common (8). Furthermore, current frameworks often disproportionately prioritize the acute deployment phase, leaving critical gaps in protocols for mission termination and “exit strategies” (16). Additionally, despite the zoonotic nature of many emerging threats, laboratory capacities remain functionally siloed; the lack of integrated veterinary testing in rapid response units severely undermines the operationalization of the “One Health” approach (17). As such, decision-makers in these settings are often forced to rely on *ad hoc* strategies, with limited empirical validation.

Therefore, this systematic review aims to: (1) Describe the roles of clinical, public health, POCT, mobile, and veterinary laboratories across pre-disaster, response, and recovery phases. (2) Identify and compare common and context-specific challenges faced by laboratory systems in HICs versus LMICs and across disaster types (natural, epidemic, human-induced). (3) Synthesize documented solutions and assess their distribution and reported effectiveness. (4) Develop an integrated analytical framework linking identified challenges with appropriate solutions to inform policy and practice.

## Methods

This qualitative evidence synthesis was conducted and reported in accordance with Preferred Reporting Items for Systematic Reviews and Meta-Analyses (PRISMA) 2020 (18). Methodological decisions were guided by the Joanna Briggs Institute (JBI) Manual for Evidence Synthesis (2023) (19). The protocol was prospectively registered in PROSPERO (CRD420251053068).

### Search method

Two reviewers, TTK and NML, searched the PubMed, Scopus, and Embase databases for relevant literature in May 2025. The search was restricted to full-text articles published within the last 25 years. A literature search was conducted using keywords based on the population, exposure, comparison, and outcome (PECO) framework. The research question for this systematic review was defined using the PECO framework as follows: Population (P): Medical laboratory infrastructures and modalities, including laboratory systems & networks, mobile, veterinary, point-of-care (POCT), clinical, and public health laboratories, involved in disaster medicine; Exposure (E): Disaster or emergency scenarios, such as natural catastrophes, man-made crises, or epidemic/pandemic outbreaks; Comparison (C): No comparator was required, as this review uses qualitative synthesis; Outcomes (O): Roles and core functions, preparedness measures, response activities, technical and operational capabilities, challenges, and lessons learned for laboratories in disaster medicine. The terms were “laboratory,” “public health emergency,” “preparedness,” and “response.” Boolean operators “AND” and “OR” were used to facilitate the search. Additionally, a combination of keywords was used in the title, abstract, full text, and Medical Subject Headings (MeSH). Reference lists of the identified publications were also examined for additional relevant studies.

### Eligibility criteria

Studies were eligible if they involved any type of laboratory system (clinical, public health, POCT, mobile, or veterinary) participating in disaster or major-incident responses. Eligibility was restricted to primary qualitative, case, and mixed-methods studies with extractable qualitative data, published from 1 January 2000 to 8 July 2025. Studies published in other languages were listed but excluded from data extraction and synthesis.

### Data extraction

All search results were imported into Rayyan (20). Duplicate records were removed using both automated and manual procedures. Title and abstract screening, followed by full-text review, was independently conducted by two reviewers using predefined eligibility criteria. Discrepancies were resolved through discussion or, when necessary, adjudication by a third reviewer. Reasons for study exclusion at the full-text screening stage were documented in full. Data were extracted independently by two reviewers using a piloted Microsoft Excel form. Extracted information included bibliometric details, disaster type, laboratory type, study design, and all verbatim content relevant to laboratory roles, challenges, and proposed solutions.

### Quality appraisal

The methodological quality of included studies was appraised using the JBI Critical Appraisal Checklists appropriate to each study design (16). Two reviewers independently rated each checklist item as Yes/No/Unclear/Not applicable (NA). For summary purposes, items were coded Yes = 1; No or Unclear = 0, and NA items were excluded from the denominator. A percentage quality score was calculated for each study as: score (%) = (sum of Yes or number of applicable items) × 100. Studies were classified *a priori* as High (≥75%), Moderate (50–74%), or Low (<50%).

### Data synthesis

Data synthesis followed the meta-aggregation approach recommended by the JBI (19). The detailed process description of the three-stage coding process is provided in the S2 File. Each meta-theme was assessed using the Grading of Recommendations Assessment, Development and Evaluation - Confidence in the Evidence from Reviews of Qualitative research approach, evaluating methodological limitations, coherence, adequacy of data, and relevance to the review objective (21).

## Results

**Figure 1:**
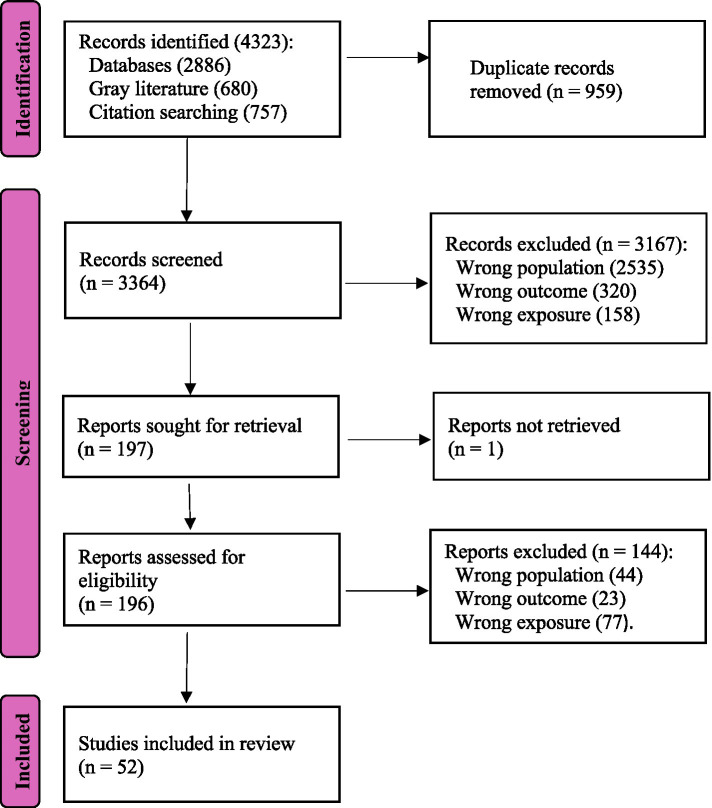
PRISMA flow diagram. The diagram illustrates the flow of information through the different phases of the systematic review, including identification, screening, and inclusion of studies.

The search yielded a total of 4,323 records, including 2,886 from databases (PubMed = 853, Scopus = 1,223, Embase = 810) and 1,437 from other sources (gray literature = 680, snowballing = 757). After removing 959 duplicates, 3,363 records were screened. Following title and abstract screening, 196 full-text articles were assessed for eligibility, of which 52 met the inclusion criteria for synthesis (Figure 1).

### Characteristics of included studies

Using the adapted JBI appraisal tool, 34 studies were rated as high quality (20–53), 13 studies as medium (14, 54–65), and 5 studies as low (66–70). Quality appraisal results by study type and setting are summarized in Table 1 and presented in full in S3 File.

**Table 1:** Characteristics of the 52 studies included in the systematic review.

Author and year	Type of design	Country, income level	Type of laboratory	Main disaster group	Type of disaster	Quality of evidence
Nia Clements et al., 2024 (54)	Qualitative research	United States, HICs	Veterinary	Epidemic	COVID-19	Medium
Tara K. Sealy et al., 2016 (22)	Descriptive study	West Africa (Guinea, Liberia, Sierra Leone) and United States, HICs, LMICs	Clinical, Public Health	Epidemic	Ebola Virus	Low
SM Rashed Ul Islam et al., 2021 (23)	Case report	Bangladesh, LMICs	Clinical	Epidemic	COVID-19	High
Julie Villanueva et al., 2019 (24)	Case Series	United States, HICs	Public Health	Epidemic	Ebola Virus, MERS-CoV, ZIKA Virus	High
Ji-Rong Yang et al., 2017 (25)	Case Report	Taiwan, HICs	Public Health	Epidemic	Novel Influenza Virus	High
Tricia A. Aden et al., 2022 (55)	Case Series	United States, HICs	Veterinary, Public Health	Epidemic	Monkeypox	High
Issa Abu-Dayyeh et al., 2023 (26)	Case Report	Jordan, LMICs	Clinical, Public Health	Epidemic	COVID-19	Medium
Michael B. Heller et al., 2002 (66)	Case Report	United States, HICs	Public Health	Human-induced	Bioterrorism	High
James M. Crawford et al., 2010 (27)	Case Report	United States, HICs	Clinical	Epidemic	Influenza A H1N1	High
Zaruhi Grigoryan et al., 2025 (67)	Descriptive study	Armenia, LMICs	Clinical, Public Health	Epidemic	COVID-19	Medium
Alicia R. Feagins et al., 2019 (56)	Descriptive study	Multiple countries in Sub-Saharan Africa (e.g., Burkina Faso, Mali, Chad, Niger, Togo), LMICs	Public Health	Epidemic	Meningitis	Medium
Giulietta Venturi et al., 2020 (28)	Descriptive study	European Union and European Economic Area countries (EVD-LabNet Network) and other member states, responding to an epidemic originating from Thailand, HICs, and LMICs	Public Health	Epidemic	Chikungunya Virus	High
R. Mögling et al., 2017 (29)	Qualitative research	European Union, HICs	Public Health	Epidemic	ZIKA Virus	Low
A. Mérens et al., 2012 (30)	Case Series	France, HICs	Clinical, Veterinary, Public Health	Human-induced	Bioterrorism	High
Chantal B. E. M. Reusken et al., 2020 (31)	Qualitative research	European Union, HICs	Public Health	Epidemic	COVID-19	Low
MAJ Julie A. Pavlin et al., 2000 (32)	Descriptive study	United States, HICs	Public Health	Human-induced	Bioterrorism	High
Kevin Taylor et al., 2014 (33)	Case Report	New Zealand, HICs	Clinical	Natural	Earthquake	High
Mona Mahmoud et al., 2022 (34)	Descriptive study	United Arab Emirates, HICs	Public Health	Epidemic	Avian influenza, and MERS-CoV	High
Fred Rodriguez et al., 2018 (35)	Descriptive study	United States, HICs	Clinical	Natural	Hurricane, Flood	High
Emmie de Wit et al., 2016 (36)	Case Report	Liberia, LMICs	Clinical	Epidemic	Ebola Virus	Medium
Jimmy N. Yu et al., 2010 (37)	Descriptive study	Haiti, LMICs	Public Health	Natural	Earthquake	Medium
Tracie EisBrenner et al., 2020 (57)	Descriptive study	Canada, HICs	Public Health	Epidemic	COVID-19	High
Nils Stanislawski et al., 2023 (58)	Case Report	Germany, HICs	Public Health	Epidemic	COVID-19	High
Stephen B. Kennedy et al., 2016 (38)	Case Report	Liberia, LMICs	Public Health	Epidemic	Ebola Virus	High
Florian Gehre et al., 2024 (59)	Case Report	East African Community (EAC): Burundi, Kenya, Rwanda, Tanzania, South Sudan, Uganda, and Democratic Republic of Congo (DRC), LMICs	Public Health	Epidemic	Monkeypox	High
Andrew W. Bartlow et al., 2024 (60)	Case Report	Uganda, LMICs	Public Health	Epidemic	COVID-19, Ebola Virus	High
Nooshafarin Safadel et al., 2024 (61)	Case Report	Iran, LMICs	Public Health	Epidemic	COVID-19	High
Qizhi Diao et al., 2024 (68)	Case Report	China, LMICs	Clinical	Epidemic	COVID-19	High
Parvathy Krishnan Krishnakumari et al., 2024 (39)	Case Report	Nepal, LMICs	Clinical, Public Health	Epidemic	COVID-19	Medium
You La Jeon et al., 2023 (40)	Case Series	South Korea, HICs	Clinical	Epidemic	COVID-19	High
James Alan Donnelly et al., 2023 (41)	Case Report	Ireland, HICs	Clinical	Epidemic	COVID-19	High
Ali A. Al-Waleedi et al., 2023 (42)	Descriptive study	Yemen, a LMICs	Public Health	Epidemic, Human-induced	Novel Influenza Virus, Conflict, and Complex Emergency	High
Florian Gehre et al., 2023 (43)	Case Report	East African Community (EAC): Tanzania, Uganda, Kenya, Burundi, Rwanda, South Sudan, and the Democratic Republic of Congo, LMICs	Public Health	Epidemic	Monkeypox	High
Daniel Mukadi-Bamuleka et al., 2023 (62)	Descriptive study	Democratic Republic of Congo, LMICs	Public Health	Epidemic	Ebola Virus	Medium
Philip Bacchus et al., 2021 (69)	Case Report	Sweden, HICs	Public Health	Epidemic	COVID-19	High
Reynolds Salerno et al., 2020 (44)	Case Report	United States, HICs	Clinical, Public Health	Epidemic	ZIKA Virus	High
Bassirou Diarra et al., 2022 (45)	Case Report	Mali, LMICs	Public Health	Epidemic	COVID-19	Medium
Heather P. McLaughlin et al., 2021 (63)	Case Report	United States, HICs	Public Health	Epidemic	COVID-19	High
Denise Toney et al., 2021 (64)	Qualitative research	United States, HICs	Public Health	Epidemic	COVID-19	High
Harimat Hendarwan et al., 2020 (65)	Qualitative research	Indonesia, LMICs	Public Health	Epidemic	COVID-19	Medium
Abdourahamane Yacouba et al., 2020 (46)	Case Report	Niger, LMICs	Public Health	Epidemic	COVID-19	Low
Frantz Jean Louis et al., 2017 (47)	Descriptive study	Haiti, LMICs	Public Health	Natural	Earthquake	High
Victoria Katawera et al., 2019 (48)	Descriptive study	Liberia, LMICs	Public Health	Epidemic	Ebola Virus	Medium
Jeniffer Concepción-Acevedo et al., 2018 (49)	Descriptive study	United States, HICs	Public Health	Natural	Hurricane	High
Sun Y et al., 2016 (70)	Case Report	Sierra Leone, LMICs	Public Health	Epidemic	Ebola Virus	High
Erika Balfour et al., 2016 (50)	Case Series	United States, HICs	Clinical	Human-induced, Epidemic, Natural	Hurricane, Influenza A H1N1, Fire, Flood	High
R Wölfel et al., 2015 (51)	Descriptive study	Multiple West African countries (Guinea, Nigeria, Liberia, Sierra Leone), LMICs	Public Health	Epidemic	Ebola Virus	Medium
Randall T. Hayden et al., 2010 (74)	Qualitative research	United States, HICs	Clinical	Epidemic	Influenza A H1N1	Low
Judith L. Isaac-Renton et al., 2012 (52)	Descriptive study	Canada, HICs	Public Health	Epidemic	Influenza A H1N1	High
Sheena Adamson et al., 2010 (53)	Descriptive study	Australia, HICs	Clinical, Public Health	Epidemic	Influenza A H1N1	High
Christopher S. Sandlin et al., 2009 (72)	Descriptive study	United States, HICs	Public Health	Human-induced	Chemical Terrorism	High
Patricia A. Nolan et al., 2003 (73)	Case Series	United States, HICs	Public Health	Human-induced	Bioterrorism	Medium

HICs, high-income countries; LMICs, low- and middle-income countries; COVID-19, Coronavirus disease 2019; MERS-CoV, middle east respiratory syndrome Coronavirus; EVD, Ebola virus disease.

### Core roles of the laboratory system across the disaster timeline

The central role of laboratory systems in disaster management was consistently identified across all 52 included studies (34 high-quality, 13 medium-quality, and 5 low-quality). Rather than functioning purely reactively, laboratories were described as evolving operational entities whose roles adapt across the disaster timeline. To better conceptualize this, findings were categorized by disaster phase: preparedness (pre-disaster), acute response (during-disaster), and recovery/enhancement (post-disaster), reflecting a continuum of laboratory engagement throughout emergency cycles (71).

### Pre-disaster: building the safety net

Data from of 42 studies (32 high-quality, 7 medium-quality, and 3 low-quality) described laboratory preparedness functions spanning surveillance, protocol development, and operational readiness. Public-health and veterinary laboratories were frequently noted as forming the “surveillance spine,” conducting integrated monitoring and modeling of zoonotic spill-over risks (25, 30, 42, 43, 45–47, 54, 55, 59, 64, 67). Clinical laboratories were reported to refine and validate standard operating procedures (SOPs) through routine system-wide drills, enhancing staff readiness and procedural consistency (30, 31, 36, 41, 47, 50, 52, 58, 59, 61, 63, 68–70, 72, 73). Regarding mobile assets, findings indicated that preparedness mainly involved the logistical staging of RRMLs and the stockpiling of reagents to ensure these operational extensions could be deployed immediately upon activation (37–39, 43, 45, 47, 51, 58, 59, 62, 69, 70). This constellation of preparatory activities was seen to establish a scalable diagnostic backbone capable of rapid activation within hours of an event (22, 27, 30, 32, 40, 41, 43, 55, 59, 61, 63, 66, 67).

### During-disaster: rapid, layered diagnostics

During the acute response phase, findings from 46 studies (30 high-quality, 11 medium-quality, and 5 low-quality) illustrated how a multi-tiered laboratory network facilitated rapid diagnostics and resource optimization. Public-health laboratories were frequently characterized as frontline sentinel systems, scaling outbreak detection and surveillance capacity in real time (24, 25, 27, 32, 50, 53, 56, 58, 64, 66, 72–74). Clinical laboratories providing timely diagnostic support crucial for effective medical decision-making (22, 27, 35, 40, 41, 50, 53, 67). Veterinary laboratories were observed supporting this network by implementing reagent-sparing protocols, such as sample pooling (37, 41, 54, 74). Notably, the studies described RRMLs and POCT not as independent silos, but as flexible operational modalities deployed to extend the reach of the central system. These forward-deployed units provided critical diagnostic surge capacity in resource-limited or inaccessible zones, reducing turnaround times to under 20 min for critical analytes (26, 46, 48, 51, 58, 59, 62, 63, 65, 69).

### Post-disaster: recovery and system strengthening

Post-disaster activities were discussed in 38 studies (20 high-quality, 13 medium-quality, and 5 low-quality), with an emphasis on restoring core functions and translating operational lessons into future preparedness. Public-health laboratories were described as leading after-action quality reviews, revising contingency plans and surveillance frameworks based on response performance (26, 27, 33, 38, 57, 61, 63, 64, 66, 67, 69). Clinical laboratories focused on clearing test backlogs and reestablishing routine diagnostic services (35, 40, 41, 46–48, 50, 57, 63, 67, 74). A subset of studies specifically highlighted the “exit strategy” for deployed assets, where data from POCT and RRML missions were analyzed to refine future deployment algorithms and training curricula (25, 37, 38, 41, 47). These actions were interpreted as serving dual goals: meeting immediate recovery demands and reinforcing long-term system resilience (37, 50, 74).

### Cross-cutting: network architecture and interoperability

Across all phases, the laboratory network operated on a hub-and-spoke model: the static analytical backbone is formed by the integration of public health, veterinary, and reference clinical laboratories. These institutions function as the central nodes for policy, confirmatory testing, and data aggregation as central hubs, while complementing this core, the Surge Capacity Layer, comprising deployed RRMLs and peripheral POCT platforms, functions as the flexible “spokes,” extending diagnostic reach to the crisis epicenter (25, 30, 43, 45, 47, 58, 59, 62, 63, 65, 69). This bi-directional architecture enabled responsive information exchange and adaptive resource mobilization across the system. However, limitations in interoperability, such as mismatched data standards or siloed information systems, emerged as a consistent barrier, linking directly to broader operational challenges addressed in Theme 2.

### Challenges and solutions

A cross-contextual synthesis of the 52 included studies identified seven recurrent challenge domains (T1–T7) that consistently undermined laboratory system resilience across all phases of disaster management. These were derived from 274 primary codes grouped during the meta-aggregation process presented in the S4 File. Corresponding to these threats, seven solution clusters (G1–G7) were identified based on multi-study reporting and associated improvements in laboratory performance (Table 2).

**Table 2:** Cross-domain challenges and corresponding solutions for laboratory system resilience.

Challenge domain (T)	Description	Corresponding solution (G)	Description
T1. Infrastructure and logistics	Structural fragility, limited mobility, and disrupted utilities hinder laboratory operations.	G1. Redundant infrastructure and mobile deployments	Ensure operational continuity through backup facilities and mobile laboratories.
T2. Supply chain instability	Delayed deliveries, limited suppliers, and stockouts compromise resource availability.	G2. Diversified sourcing and stockpiling strategies	Stabilize inputs via multiple suppliers and strategic reserves.
T3. Workforce constraints	Surge capacity gaps, training deficiencies, and staff attrition affect human resource availability.	G3. Workforce surge mechanisms and cross-training programs	Address workforce shortages through scalable staffing and skill versatility.
T4. Quality, biosafety, and chain of custody	Lapses in SOPs, EQA, and contamination control jeopardize diagnostic integrity.	G4. Adoption of EQA schemes and harmonized SOPs	Uphold diagnostic standards and biosafety through harmonization and external validation.
T5. Administrative and regulatory barriers	Licensing delays and fragmented frameworks impede timely operations.	G5. Proactive regulatory navigation and platform standardization	Reduce bureaucratic delays through early engagement and standardization.
T6. IT and data interoperability	Incompatible systems and fragmented information flows disrupt data management.	G6. Middleware integration and centralized LIMS	Enhance data continuity via integrated digital systems.
T7. Cybersecurity vulnerabilities	Threats to data integrity, network access, and system uptime expose critical risks.	G7. Secure, access-controlled digital networks	Safeguard laboratory information infrastructure through controlled access and security protocols.

SOPs, standard operating procedures; EQA, external quality assessment; LIMS, laboratory information management system; IT, information technology.

### Overarching cross-cutting challenge clusters

Across all disaster contexts, synthesis of 35 studies (31 high-quality, 1 medium-quality, and 3 low-quality) identified five universally recurring challenges: infrastructure and logistics fragility (T1), supply chain instability (T2), workforce limitations (T3), quality and biosafety pressures (challenges in keeping tests accurate and safe in emergencies) (T4), and fragmented data interoperability (difficulties in sharing and integrating data across disconnected systems during crises) (T6) (22, 23, 26, 33, 35, 38, 42, 50, 51, 54, 57, 58, 60–62, 64–66). In high-income settings, two additional clusters emerged: administrative-regulatory friction (T5) related to dual-use export controls, and increasing cybersecurity exposure (T7). Corresponding solution clusters (G1–G7) were identified in both HIC and LMIC settings, with context-specific adaptations detailed below (23, 29, 38, 54, 58–60, 66–68).

### The tri-axial challenge landscape

Synthesis of the 52 included studies revealed that laboratory challenges are not merely geographical but structural. Findings were categorized into three distinct operational axes: Scarcity, Complexity, and Security.

#### The Scarcity Axis: infrastructure and resource autonomy

The Scarcity Axis, observed most clearly in LMIC outbreaks and natural disasters (23 studies), describes settings where the primary vulnerability is the inability to maintain operational autonomy under shock. Reagent and kit volatility (T2), driven by unpredictable donor procurement cycles and customs clearance delays, critically hampered outbreak response (22, 23, 38, 39, 42, 45, 46, 51, 56, 60, 62, 65, 67). During natural disasters, physical isolation due to infrastructure collapse (T1), for example, flood-damaged transport corridors, further amplified supply-chain breakage and peripheral stockouts (37). Workforce limitations (T3) compounded these structural failures; many affected regions depended on modular “train-the-trainer” programs and the deployment of simplified point-of-care testing (POCT) devices, both of which buffered the shortage of specialist personnel and enabled minimal-function service continuity (G3) (22, 23, 26, 38, 43, 45, 48, 56, 59, 61, 65, 67).

In these constrained settings, survival hinged on restoring autonomy through containerized laboratories with independent power and water systems (G1), the rapid development and validation of local diagnostic assays alongside regional supplier diversification (G2), pre-positioned reagent reserves in disaster-prone regions (G2), and workforce-extending strategies such as training cascades and POCT simplification (G3) (23, 38, 45, 46, 48, 51, 59, 61, 65, 67, 70).

#### The Complexity Axis: data fragmentation and standardization

By contrast, the Complexity Axis, dominant in high-income settings and large-scale epidemics (22 studies), reflects systems where the challenge lies not in resource scarcity but in coordinating heterogeneous platforms and fragmented data streams. Information system fragmentation (T6) was the most pervasive barrier, as inconsistent Laboratory Information Management System (LIMS) architectures and proprietary assay platforms led to duplicate ordering and reporting delayed reporting, and impaired situational awareness (22, 27–29, 41, 44, 50, 54, 58, 63, 64). Simultaneously, rapid diagnostic scale-up introduced cross-platform quality pressures (T4), intensifying the need for consistent interpretation across diverse technologies.

Mitigation in these settings focused on harmonization and integration: Deployment of Health Level Seven International/Fast Healthcare Interoperability Resources middleware and raw-data access agreements to limit manual reconciliation (G6); adoption of EQA schemes and harmonized SOPs to standardize performance across diverse platforms (G4); and use of vendor diversification or limited on-shoring (G2) not to counter T2 scarcity but to increase redundancy and reduce dependence on single-platform bottlenecks (22, 27, 41, 44, 53, 57, 58, 63, 64, 75). Following large-scale natural disasters, additional coordination failures arose from incompatible event-logging schemas, requiring pre-agreed data-sharing protocols and mobile communication hubs to re-establish multi-agency data flow (33, 35, 49, 50).

#### The Security Axis: legal and biological integrity

The Security Axis, most pronounced in conflict settings and human-induced disasters (six studies), encompasses contexts where legal, administrative, and security pressures dominate system vulnerability. Dual-use export controls (T5) substantially prolonged procurement of essential diagnostic materials (30, 32, 66, 72). Conflict-affected settings imposed strict requirements for biosafety and chain-of-custody integrity (T4), while increased digitization exposed laboratories to cybersecurity threats (T7) (30, 32, 66, 72, 73).

Systems operating under this axis employed proactive security-focused mitigation: early licensing and the adoption of closed, low-bio-risk platforms to minimize regulatory delays (G5); digital chain-of-custody ledgers to increase evidentiary defensibility (G6); and secure site-to-site VPN architectures to protect data and operational continuity (G7) (30, 32, 66, 72, 73).

## Discussion

### Integrated surveillance and network interoperability model (ISNIM)

During line-by-line coding and thematic synthesis of 52 studies, distinct functional clusters emerged, yet their interaction revealed critical operational gaps: (1) The National Core Layer: Public Health, Clinical, and Veterinary laboratories into a unified central governance pillar. This triad forms the static analytical backbone, responsible for aggregating large-scale testing volumes and, crucially, conducting integrated zoonotic risk modeling at the source; and (2) The Surge Capacity Layer: Comprising RRMLs and POCT platforms, this layer is defined as a standardized, autonomous infrastructure. These units are deployed to provide diagnostic surge capacity in resource-limited or cut-off zones, strictly adhering to operational self-sufficiency standards. Unlike the Centers for Disease Control and Prevention’s Laboratory Response Network or the WHO’s International Health Regulations (IHR) core capacities, which emphasize fixed-site public health hubs, the ISNIM complements these models by embedding a field-validated POCT/mobile layer and a One Health veterinary strand. This addition addresses persistent operational gaps in rapid diagnostics and multisector early warning, particularly in low-resource or decentralized settings (13, 30, 34, 37, 38, 41, 43, 45, 46, 48, 51, 54, 55, 58, 59, 65, 67, 69, 76).

Public-health laboratories were described as forming the analytical backbone across disaster phases. Prior to emergencies, they integrated event-based, syndromic, and genomic surveillance streams and conducted joint risk assessments with veterinary counterparts. During surge phases, they coordinated assay deployment and national-scale data flow. In recovery, they led after-action reviews and revisions of preparedness protocols (22, 24, 25, 32, 38, 40, 42, 46, 52, 54, 55, 59–64, 66, 69, 72). Clinical laboratories were reported to complement these functions by refining SOPs during preparedness and delivering high-throughput diagnostics during response (27, 33, 40, 46, 48, 55, 57, 61, 65). For example, they were responsible for 85.6% of all COVID-19 tests under South Korea’s national testing policy, a figure drawn from official government documentation (40). Veterinary laboratories provided One Health early warning through zoonotic spillover monitoring and sentinel testing, contributing genomic data that refined public health risk appraisals (30, 36, 54, 55, 60). These role patterns closely reflect the mandates of CLSI GP36-A, which positions clinical and public-health laboratories as core actors in preparedness, continuity, and coordinated emergency operations (8). They also align with the I2SL resilience framework, which emphasizes redundancy and protected infrastructure consistent with the backbone, surge architecture observed (10). Finally, the cross-sectoral surveillance contributions of veterinary laboratories parallel the One Health Zoonoses Guide, although operational integration remains limited in practice (11).

The Surge Capacity layer extended system reach and contextual adaptability. POCT units furnish ultra rapid (< 20 min) triage at isolation wards, field clinics, and border points, thereby relieving hub bottlenecks and improving patient flow (8, 9, 22, 26, 40, 41). Mobile or modular laboratories, typically containerized biosafety level (BSL) 2/3 units with autonomous utilities, delivered onsite PCR/ELISA in remote or cut-off areas with turnaround times as short as 4 hours, and were later redeployed for post-event surveillance (22, 36, 39, 51, 58, 59, 65, 69). Their operational profile closely aligns with the MOST minimum standards for RRML deployment and the GOARN Diagnostic Surge Capacities framework, which emphasizes coordinated, interoperable field diagnostics integrated into national emergency response structures (12, 15). Likewise, the multisectoral mobilization of public-health, veterinary, and clinical laboratories observed in this review aligns with the Tripartite One Health guidance, which underscores coordinated surveillance, shared risk assessment, and unified technical action across the human–animal–environment interface as core principles for national emergency systems (11). However, a notable scarcity of qualitative data regarding the long-term sustainability and cross-sectoral integration of these assets was observed across the 52 included studies. Specifically, while deployment logistics were well-documented, descriptions of ‘end of mission’ protocols were largely absent. This finding is consistent with Mushasha et al., who reported that standardized decommissioning frameworks remain underdeveloped (16). Similarly, concrete operational descriptions of veterinary testing within mobile units were scarce in our dataset. This observation aligns with the quantitative asymmetry identified by Trojnacki et al., who found that only 4–19% of mobile laboratories in Africa and Europe possess the actual capacity to process animal samples, limiting the realization of the One Health framework (17).

### Operational axes

Based on the synthesis findings, laboratory challenges were found to align along a three-tiered axis: Scarcity (characterized by limited resources, fragile infrastructure, and workforce constraints in LMICs), Complexity (driven by technological heterogeneity and procedural intricacies in HICs), and Security (reflecting biosafety, regulatory, and resilience challenges in conflict or CBRN-prepared settings). Each gradient was associated with distinct challenge clusters (e.g., T1–T3 for Scarcity; T4, T6 for Complexity; T4, T5, T7 for Security) and corresponding solution strategies (G1–G7). These patterns are summarized in Table 3.

**Table 3:** Gradient of laboratory challenges and solutions by contextual setting.

Context	Axis	Associated challenge domains (T)	Key solutions (G)	Guideline alignment (MOST – GOARN – One Health)	Additional notes
Low- and Middle-Income Countries (LMICs)	Scarcity – resource limitations	T1: Infrastructure fragility T2: Volatile reagent supply T3: Limited workforce capacity	G1: Mobile/modular labs G2: Diversified sourcing G3: Train-the-trainer programs	MOST OSL 8.7: Autonomy of RRMLs (power/water for 14 days) MOST QMS 18: Competency maintenance GOARN DiSC: Rapid surge deployment modules	Focused on basic operational continuity and local workforce scaling
High-Income Countries (HICs)	Complexity – integration and quality assurance	T4: Multi-platform quality/biosafety T6: Data-layer fragmentation	G4: Platform standardization G6: Middleware and LIMS integration	MOST KIM 24.3: Data compatibility & open API QMS 20: Equipment oversight I2SL Resilience Guide: Redundant data & infrastructure continuity	Emphasis on interoperability and cross-system validation
Conflict-Affected or CBRN-Prepared Environments (CCE)	Security – regulatory and cyber-bio risk	T4: Chain-of-custody and biosafety T5: Dual-use regulatory barriers T7: Cybersecurity threats	G5: Early regulatory navigation G6: Secure digital platforms G7: Site-to-site VPNs (Virtual Private Network) and access-controlled systems	MOST OCI 7: Custodianship & Nagoya compliance KIM 26: Cybersecurity standards GOARN: Secure, interoperable mission deployment	Cybersecurity risks (T7) were addressed via network-level solutions (VPNs)
Cross-Cutting Enablers		T1-related disruptions in all settings	G1: Mobile labs/POCT systems	MOST modular architecture & GOARN surge model enable flexible deployment; One Health TZG supports multisectoral links	Served as continuity bridges across diverse disaster settings and resource levels

POCT, point-of-care testing; VPNs, virtual private networks; CBRN, chemical, biological, radiological, and nuclear; HICs, high-income countries; LMICs, Low- and middle-income countries; RRML, rapid response mobile laboratory; LIMS, laboratory information management system; MOST, minimum operational standards and typology; GOARN DiSC, global outbreak alert and response network – diagnostic surge capacities; TZG, tripartite zoonoses guide; MTA, material transfer agreement; COC, chain of custody; QMS, quality management system; KIM, key interoperability measures; OCI, operational custodianship and integrity.

Benchmarking against four foundational laboratory guidelines, namely the WHO-RRML, WHO Public Health Emergency Laboratories (PHE-Labs) Manual, the Laboratory Resilience Best Practices (LRBP) Guide, and Clinical and Laboratory Standards Institute (CLSI) GP36-A, demonstrated that the Scarcity–Complexity–Security gradient derived from this synthesis is largely congruent with existing policy orientations, with each document providing additional, context-specific strategies for six operational scenarios (8–10, 13). Alignment with the WHO MOST (Minimum Operational Standards and Typology) and the GOARN DiSC guidance further reinforces this convergence: MOST underscores modularity, operational autonomy, and competency maintenance as core mechanisms for mitigating scarcity, while GOARN emphasizes interoperable surge deployments and coordinated field diagnostics that mirror the surge-layer patterns observed in our review (12, 15). Likewise, the Tripartite One Health Zoonoses Guide strengthens the Complexity and Security axes by highlighting multisectoral surveillance, joint risk assessments, and integrated human–animal–environment data flows, elements reflected in the contributions of veterinary laboratories within the included studies (11). Specifically, the WHO-RRML emphasizes the role of modular laboratories and train-the-trainer programs to counteract scarcity in resource-limited contexts (13). The CLSI GP36-A outlines cross-platform quality assurance standards to address data and diagnostic complexity (8). The LRBP Guide advocates for flexible architectural designs and offers an initial framework for cybersecurity preparedness (10). Finally, the WHO PHE-Labs Manual reinforces the Security layer through detailed protocols on chain-of-custody, biosafety practices, and regulation of select agents (9). This triangulation collectively substantiates the proposed ISNIM Maturity Framework (presented in the following section), while also underscoring the unaddressed T7 domain, thereby affirming the necessity of explicit cybersecurity metrics in forthcoming laboratory assessment frameworks (8, 10, 37).

### The ISNIM Maturity Framework for disaster-ready laboratories

The construction of the ISNIM Maturity Framework is grounded in findings synthesized from the 52 included studies, which consistently organized laboratory challenges along three operational axes: Scarcity (reflects fragile infrastructure and workforce gaps limiting continuity in LMICs), Complexity (denotes integration and quality-assurance demands in advanced systems) and Security (captures biosafety, regulatory, and cyber-resilience requirements in conflict or CBRN settings). To operationalize these findings, the framework integrates six authoritative guidelines, specifically prioritizing WHO MOST (for technical standards) and GOARN DiSC (for governance), alongside WHO-RRML, WHO PHE-Labs, LRBP, and CLSI GP36-A (8–10, 12, 13, 15).

The ISNIM Framework defines five maturity levels based on the specific resolution of the three axes. Levels 1–2 (Fragmented to Reactive), Characterized by dependency on local infrastructure, addressing the Scarcity Axis through basic resource reinforcement. Level 3 (Autonomous – The Trigger Point), Defined by the achievement of Operational Self-Sufficiency (meeting WHO MOST OSL 8.7). This is the critical threshold for a functional national asset; failure to maintain this level serves as a trigger for requesting international assistance. Levels 4–5 (Integrated to Resilient), Address the Complexity and Security Axes by implementing Interoperability (WHO MOST KIM 24.3) and Global Partnership mechanisms (GOARN DiSC), enabling the laboratory to function as a node in the global response network. A summary of these definitions and core capabilities is provided in Table 4.

**Table 4:** The capability maturity model for disaster-ready laboratories (CMM-5).

Level	Status	Scarcity axis – logistics & autonomy (MOST OSL)	Complexity axis – data & connectivity (MOST KIM)	Security axis – legal, biosafety & one health (MOST OCI/B&B)	International activation mechanism (GOARN DiSC)
1	Fragmented	Parasitic: Fully dependent on local power/water; collapses easily (violates OSL 8.7).	Manual: Paper or verbal reporting; no digital data (Data Void).	Ad hoc: No biosafety procedures; no veterinary/One Health mechanisms.	Recipient-only: Requires full external assistance; cannot integrate international teams.
2	Reactive	Partial: Generator available but insufficient fuel (<3 days); supply chains unreliable.	Silos: Personal Excel/Word files; data locked in isolated workstations.	Basic: Minimal biosafety; unresolved legal barriers to specimen handling.	Limited: Can receive donated supplies but lacks structures for personnel coordination.
3	Autonomous	Standardized: Meets OSL 8.7 (14-day autonomy); independent of local infrastructure.	Digitized: Local LIMS; periodic reporting via email/soft files.	Compliant: Standardized BSL-2/3; early passive veterinary surveillance.	Trigger Level: Minimum threshold for a national lab; overload triggers GOARN request.
4	Interoperable	Sustainable: Strategic stockpiles; retreat/demobilization plan aligns with OSL 11.	Connected: Implements minimum dataset (KIM 24.3); two-way data sharing with national hubs.	Coordinated: Integrated One Health surveillance; pre-approved legal frameworks (MTA).	Compatible: Capable of hosting, integrating, and commanding international mobile lab teams.
5	Resilient	Adaptive: Flexible supply chains; “zero-footprint” withdrawal strategy.	Real-time: Open APIs feeding directly into national dashboards; big-data analytics.	Ecosystem: Full Nagoya compliance (OCI 7); advanced cybersecurity (KIM 26).	Partner: Meets GOARN partner-level criteria; able to deploy support to other countries.

OSL, operational support and logistics; LIMS, Laboratory information management system; BSL, biosafety level; MTA, material transfer agreement; API, application programming interface; GOARN DiSC, global outbreak alert and response network – diagnostic surge capacities; One Health B&B, biosafety and biosecurity under the one health framework; OCI, operational custodianship and integrity; KIM, key interoperability measures; RRML, rapid response mobile laboratory.

The intended use of the model spans multiple system levels. It serves as: (1) A tool for self-assessment and improvement planning by hospital and public-health laboratories; (2) A national benchmarking reference for IHR (2005) Core Capacity 2 reporting (77); (3) A framework for donors to structure grant-making or technical assistance by verifiable advancement tiers; (4) An optional component for integration into ISO 15189 or BSL accreditation audits by adding disaster-readiness criteria (78); (5) A curriculum map for training programs, aligning competencies with maturity levels.

Progression across the ISNIM Framework is intended to be incremental and evidence-driven. Laboratories should prioritize interventions that unlock the next level of maturity: Transition to Level 3 (Autonomy): Focuses on logistics, such as installing uninterruptible power supplies and securing independent supply chains to meet MOST OSL 8.7. Transition to Level 4 (Connectivity): Focuses on digital integration, piloting open-source LIMS and adopting Minimum Data Sets (MOST KIM 24.3) to bridge data silos. Transition to Level 5 (Resilience): Involves advanced governance, such as establishing digital Chain of Custody, pre-agreed Material Transfer Agreements (MOST OCI 7), and integrated cyber-resilience exercises (28, 30, 33, 37, 38, 40, 41, 44, 45, 47, 50–52, 56, 58, 60, 62, 66, 67, 69, 72).

### Limitations

Only a few studies examined non-epidemic disasters in LMICs. More context-specific primary research is needed to validate proposed frameworks and interventions across a broader range of disaster types. Furthermore, a significant portion of the included literature was published during the COVID-19 pandemic. Unlike acute, sudden-onset disasters (e.g., earthquakes, flash floods, or chemical incidents) which require immediate mobilization within hours and typically have a limited duration, the COVID-19 pandemic represented a ‘chronic’ crisis spanning several years. Consequently, some identified solutions, such as the gradual scaling of molecular testing infrastructure or long-term workforce training, may be less applicable to hyper-acute scenarios where speed and immediate mobility are paramount. While the core principles of resilience remain relevant, the predominance of COVID-19 data may introduce a bias toward solutions favored in prolonged biological crises. Future research should aim to balance this by focusing more on laboratory responses to short-duration, high-impact events to ensure the broader generalizability of these frameworks.

## Conclusion

This systematic review of 52 studies shows that laboratories fulfill essential roles in preparedness, response, and recovery across disaster phases. However, existing preparedness frameworks insufficiently account for laboratory system capacities, leaving critical gaps in continuity, biosafety, and digital resilience. These omissions risk undermining investments in global health security and pandemic preparedness.

To address this gap, the ISNIM Maturity Framework which organizes laboratory challenges into a gradient of Scarcity, Complexity, and Security, and defines progressive levels of capability maturity. This framework provides a structured basis for benchmarking, capacity building, and cross-country comparison.

## Data Availability

The original contributions presented in the study are included in the article/Supplementary material, further inquiries can be directed to the corresponding author.
