# Supplementary material for: The role, challenges, and solutions of laboratories in disaster medicine: a systematic review
Source: Front Public Health. 2026 Jan 13;13:1726280. doi: 10.3389/fpubh.2025.1726280 (PMC12834775; doi:10.3389/fpubh.2025.1726280)
Supplement: Supplementary file 1 [file Supplementary_file_1.docx]

**Supplementary 1:** **Information Sources and Search Strategy**

**1. Information Sources**

| Source Type | Details |
| --- | --- |
| Databases | - MEDLINE (via PubMed)   - Embase (via Embase.com)   - Scopus (via Scopus.com) |
| Grey Literature | - Websites of key organizations:       + World Health Organization (WHO)       + Centers for Disease Control and Prevention (CDC)       + Global Outbreak Alert and Response Network (GOARN)       + International Federation of Red Cross and Red Crescent Societies (IFRC)       + United Nations Office for Disaster Risk Reduction (UNDRR)       + Vietnam Ministry of Health   - OpenGrey   - ProQuest Dissertations & Theses Global |
| Additional Search Methods | - Google Scholar (the first 200 results were screened) was used to ensure saturation of grey literature, in line with the recommendations by Haddaway et al.^1^  - Screening of publicly available conference proceedings relevant to laboratory systems and health emergencies |
| Manual Searching | - Reference lists of included studies and relevant systematic reviews |
| Citation Tracking | - Forward citation tracking via MEDLINE, Embase, and Scopus |

*1. Haddaway NR, Woodcock P, Macura B, Collins A. Making literature reviews more reliable through application of lessons from systematic reviews. Conservation Biology. 2015;29(6):1596-1605. doi:10.1111/cobi.12541*

**2. Search strategy**

| Pubmed | Scopus | Embase |
| --- | --- | --- |
| ("bioterrorism"[Title/Abstract] OR "chemical terrorism"[Title/Abstract] OR  "radiological emergency"[Title/Abstract] OR "pandemic"[Title/Abstract] OR  "epidemic"[Title/Abstract] OR "infectious disease outbreak"[Title/Abstract] OR  "earthquake"[Title/Abstract] OR "flood"[Title/Abstract] OR "tsunami"[Title/Abstract] OR  "hurricane"[Title/Abstract] OR "cyclone"[Title/Abstract] OR "natural disaster"[Title/Abstract] OR  "public health emergency"[Title/Abstract] OR  "Bioterrorism"[MeSH Terms] OR "Natural Disasters"[MeSH Terms] OR  "Disease Outbreaks"[MeSH Terms]) AND ("clinical laboratory"[Title/Abstract] OR "public health laboratory"[Title/Abstract] OR  "emergency laboratory"[Title/Abstract] OR "point-of-care testing"[Title/Abstract] OR  "POCT"[Title/Abstract] OR "mobile laboratory"[Title/Abstract] OR  "veterinary laboratory"[Title/Abstract] OR "Laboratories"[MeSH Terms] OR  "Point-of-Care Systems"[MeSH Terms]  ) AND ("preparedness"[Title/Abstract] OR "response"[Title/Abstract] OR  "role"[Title/Abstract] OR "function"[Title/Abstract] OR "capability"[Title/Abstract] OR  "resilience"[Title/Abstract] OR "lessons learned"[Title/Abstract] OR  "Emergency Preparedness"[MeSH Terms] OR "Disaster Planning"[MeSH Terms] ) NOT ("review"[Publication Type] ) AND ( "2000/01/01"[Date - Publication] : "2025/05/15"[Date - Publication] ) | TITLE-ABS-KEY  ( "bioterrorism" OR "chemical terrorism" OR "public health emergency" OR "natural disaster" OR "earthquake" OR "flood" OR "tsunami" OR "cyclone" OR "hurricane" OR "pandemic" OR "disaster preparedness" OR "outbreak") AND TITLE-ABS-KEY ( "clinical laboratory" OR "diagnostic laboratory" OR "mobile laboratory" OR "point-of-care testing" OR "POCT" OR "public health laboratory" OR "emergency laboratory" OR "lab response" ) AND TITLE-ABS-KEY ( "preparedness" OR "response" OR "capability" OR "role" OR "resilience" OR "capacity") AND NOT (DOCTYPE("re")) AND PUBYEAR > 1999 AND PUBYEAR < 2026 AND ( LIMIT-TO ( SUBJAREA , "MEDI" ) OR LIMIT-TO ( SUBJAREA , "HEAL" ) ) AND ( LIMIT-TO ( DOCTYPE , "ar" ) ) | ('bioterrorism':ti,ab OR 'chemical terrorism':ti,ab OR 'public health emergency':ti,ab OR 'natural disaster':ti,ab OR 'earthquake':ti,ab OR 'flood':ti,ab OR 'tsunami':ti,ab OR 'cyclone':ti,ab OR 'hurricane':ti,ab OR 'pandemic':ti,ab OR 'bioterrorism'/exp OR 'natural disaster'/exp OR 'disease outbreak') AND ('clinical laboratory':ti,ab OR 'public health laboratory':ti,ab OR 'emergency laboratory':ti,ab OR 'point-of-care testing':ti,ab OR 'mobile laboratory':ti,ab OR 'laboratory'/exp OR 'point of care testing'/exp) AND ('preparedness':ti,ab OR 'response':ti,ab OR 'role':ti,ab OR 'capability':ti,ab OR 'emergency preparedness'/exp OR 'disaster planning'/exp) AND (2000:py OR 2001:py OR 2002:py OR 2003:py OR 2004:py OR 2005:py OR 2006:py OR 2007:py OR 2008:py OR 2009:py OR 2010:py OR 2011:py OR 2012:py OR 2013:py OR 2014:py OR 2015:py OR 2016:py OR 2017:py OR 2018:py OR 2019:py OR 2020:py OR 2021:py OR 2022:py OR 2023:py OR 2024:py OR 2025:py) NOT 'review'/it |

Press 2015: Checklist for search strategy

| PRESS 2015 Item | Assessment | Comments |
| --- | --- | --- |
| 1. Translation of the research question | Adequate | The search strategy aligns well with the PECO elements (Population, Exposure, Outcome). Comparator not applicable. |
| 2. Boolean and proximity operators | Appropriate | Boolean operators (AND, OR, NOT) are used correctly. Proximity operators (e.g., NEAR/n or ADJ/n) are applied in databases that support them (e.g., Scopus). |
| 3. Subject headings (controlled vocabulary) | Used appropriately | MeSH terms in PubMed and Emtree terms in Embase are correctly integrated alongside free-text terms. |
| 4. Text word searching (free-text terms) | Comprehensive | Relevant synonyms and related terms are searched in titles and abstracts across all databases. |
| 5. Spelling, syntax, and line structure | Correct | No syntax errors identified. Parentheses and field tags are properly used in all three database strings. |
| 6. Limits and filters | Justified | Appropriate use of filters for publication year (2000–2025). Exclusion of reviews using correct syntax in all databases. No language limits were applied at the search level, consistent with eligibility criteria. |
| 7. Overall strategy appropriateness | High | The strategy is well constructed, database-specific adaptations are correctly applied, and the terms effectively cover the scope of the review topic. |
