# Supplementary figures and images for: The role, challenges, and solutions of laboratories in disaster medicine: a systematic review
Source: Front Public Health. 2026 Jan 13;13:1726280. doi: 10.3389/fpubh.2025.1726280 (PMC12834775; doi:10.3389/fpubh.2025.1726280)

**Supplementary 2: Data synthesis**


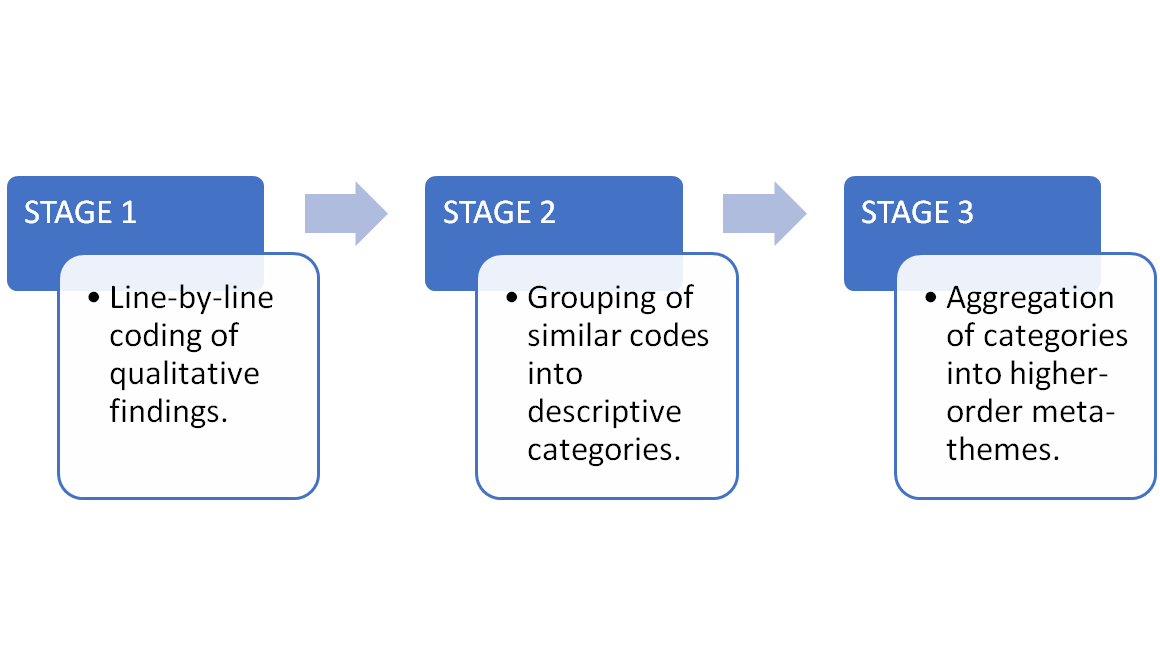

Supplement: Supplementary file 2 [file Supplementary_file_2.docx]
