# Supplementary material for: The role, challenges, and solutions of laboratories in disaster medicine: a systematic review
Source: Front Public Health. 2026 Jan 13;13:1726280. doi: 10.3389/fpubh.2025.1726280 (PMC12834775; doi:10.3389/fpubh.2025.1726280)
Supplement: Supplementary file 3 [file Supplementary_file_3.docx]

**Supplementary 3: Detailed Characteristics and Quality Appraisal of Included Studies.**

| **Author and year** | **Country, Income level** | **Type of Disaster** | **Q1** | **Q2** | **Q3** | **Q4** | **Q5** | **Q6** | **Q7** | **Q8** | **Q9** | **Q10** | **Quality** |
| --- | --- | --- | --- | --- | --- | --- | --- | --- | --- | --- | --- | --- | --- |
| Nia Clements et al, 2024 | United States, High-income | COVID-19 | Y | Y | Y | N | U | N | N | Y | Y | U | Medium |
| Tara K. Sealy et al, 2016 | West Africa & USA, High & Low-Mid income | Ebola | Y | Y | N | U | N | N | Y | N | Y | Y | Low |
| SM Rashed Ul Islam et al, 2021 | Bangladesh, Low-Mid income | COVID-19 | Y | Y | Y | Y | Y | Y | Y | Y | Y | Y | High |
| Julie Villanueva et al, 2019 | United States, High-income | Ebola, MERS-CoV, ZIKA | Y | Y | Y | Y | Y | Y | Y | Y | Y | Y | High |
| Ji-Rong Yang et al, 2017 | Taiwan, High-income | Novel Influenza | Y | Y | Y | Y | Y | Y | Y | Y | Y | Y | High |
| Tricia A. Aden et al, 2022 | United States, High-income | Monkeypox | Y | Y | Y | Y | Y | Y | Y | Y | Y | Y | High |
| Issa Abu-Dayyeh et al, 2023 | Jordan, Low-Mid income | COVID-19 | Y | Y | Y | U | Y | Y | N | Y | Y | Y | Medium |
| Michael B. Heller et al, 2002 | United States, High-income | Bioterrorism | Y | Y | Y | Y | Y | Y | Y | N | Y | Y | High |
| James M. Crawford et al, 2010 | United States, High-income | Influenza A H1N1 | Y | Y | Y | Y | Y | Y | Y | Y | Y | Y | High |
| Zaruhi Grigoryan et al, 2025 | Armenia, Low-Mid income | COVID-19 | Y | Y | Y | Y | U | Y | N | Y | Y | Y | Medium |
| Alicia R. Feagins et al, 2019 | Sub-Saharan Africa, Low-Mid | Meningitis | Y | Y | Y | Y | Y | U | Y | Y | Y | Y | Medium |
| Giulietta Venturi et al, 2020 | EU & EEA, High & Low-Mid | Chikungunya | Y | Y | Y | Y | Y | Y | Y | Y | Y | Y | High |
| R. Mögling et al, 2017 | European Union, High-income | ZIKA | Y | Y | N | N | U | N | NA | N | Y | Y | Low |
| A. Mérens et al, 2012 | France, High-income | Bioterrorism | Y | Y | Y | Y | Y | Y | Y | Y | Y | Y | High |
| C.B.E.M. Reusken et al, 2020 | European Union, High-income | COVID-19 | Y | Y | N | U | N | U | N | Y | NA | Y | Low |
| MAJ Julie A. Pavlin et al, 2000 | United States, High-income | Bioterrorism | Y | Y | Y | Y | Y | Y | Y | Y | Y | Y | High |
| Kevin Taylor et al, 2014 | New Zealand, High-income | Earthquake | Y | Y | Y | Y | Y | Y | N | Y | Y | Y | High |
| Mona Mahmoud et al, 2022 | UAE, High-income | Avian flu, MERS-CoV | Y | Y | Y | Y | Y | Y | Y | Y | Y | Y | High |
| Fred Rodriguez et al, 2018 | United States, High-income | Hurricane, Flood | Y | Y | Y | Y | Y | Y | N | Y | Y | Y | High |
| Emmie de Wit et al, 2016 | Liberia, Low-Mid income | Ebola | Y | Y | Y | Y | Y | Y | Y | N | Y | Y | Medium |
| Jimmy N. Yu et al, 2010 | Haiti, Low-Mid income | Earthquake | Y | Y | Y | N | Y | Y | U | Y | Y | Y | Medium |
| Tracie EisBrenner et al, 2020 | Canada, High-income | COVID-19 | Y | Y | Y | Y | Y | Y | Y | Y | Y | Y | High |
| Nils Stanislawski et al, 2023 | Germany, High-income | COVID-19 | Y | Y | Y | Y | Y | Y | Y | Y | Y | Y | High |
| Stephen B. Kennedy et al, 2016 | Liberia, Low-Mid income | Ebola | Y | Y | Y | Y | Y | Y | Y | Y | Y | Y | High |
| Florian Gehre et al, 2024 | EAC, Low-Mid income | Monkeypox | Y | Y | Y | Y | Y | Y | Y | Y | Y | Y | High |
| Andrew W. Bartlow et al, 2024 | Uganda, Low-Mid income | COVID-19, Ebola | Y | Y | Y | Y | Y | Y | Y | Y | Y | Y | High |
| N. Safadel et al, 2024 | Iran, Low-Mid income | COVID-19 | Y | Y | Y | Y | Y | Y | Y | Y | Y | Y | High |
| Qizhi Diao et al, 2024 | China, Low-Mid income | COVID-19 | Y | Y | Y | Y | Y | Y | Y | Y | Y | Y | High |
| P. K. Krishnakumari et al, 2024 | Nepal, Low-Mid income | COVID-19 | Y | Y | Y | Y | Y | U | Y | Y | Y | Y | Medium |
| You La Jeon et al, 2023 | South Korea, High-income | COVID-19 | Y | Y | Y | Y | Y | Y | Y | Y | Y | Y | High |
| James Alan Donnelly et al, 2023 | Ireland, High-income | COVID-19 | Y | Y | Y | Y | Y | Y | Y | Y | Y | Y | High |
| Ali A. Al-Waleedi et al, 2023 | Yemen, Low-Mid income | Novel Influenza, Conflict | Y | Y | Y | Y | Y | Y | Y | Y | Y | Y | High |
| Florian Gehre et al, 2023 | EAC, Low-Mid income | Monkeypox | Y | Y | Y | Y | Y | Y | Y | Y | Y | Y | High |
| D. Mukadi-Bamuleka et al, 2023 | DR Congo, Low-Mid income | Ebola | Y | Y | Y | Y | Y | Y | N | Y | Y | Y | Medium |
| Philip Bacchus et al, 2021 | Sweden, High-income | COVID-19 | Y | Y | Y | Y | Y | Y | Y | Y | Y | Y | High |
| Reynolds Salerno et al, 2020 | United States, High-income | ZIKA | Y | Y | Y | Y | Y | Y | Y | Y | Y | Y | High |
| Bassirou Diarra et al, 2022 | Mali, Low-Mid income | COVID-19 | Y | Y | Y | Y | Y | N | Y | Y | Y | Y | Medium |
| H. P. McLaughlin et al, 2021 | United States, High-income | COVID-19 | Y | Y | Y | Y | Y | Y | Y | Y | Y | Y | High |
| Denise Toney et al, 2021 | United States, High-income | COVID-19 | Y | Y | Y | Y | Y | Y | Y | Y | Y | Y | High |
| Harimat Hendarwan et al, 2020 | Indonesia, Low-Mid income | COVID-19 | N | Y | Y | Y | U | N | N | Y | Y | U | Medium |
| A. Yacouba et al, 2020 | Niger, Low-Mid income | COVID-19 | Y | Y | N | N | Y | N | U | N | Y | Y | Low |
| Frantz Jean Louis et al, 2017 | Haiti, Low-Mid income | Earthquake | Y | Y | Y | Y | Y | Y | Y | Y | Y | Y | High |
| Victoria Katawera et al, 2019 | Liberia, Low-Mid income | Ebola | Y | Y | Y | Y | Y | U | Y | Y | Y | Y | Medium |
| J. C.-Acevedo et al, 2018 | United States, High-income | Hurricane | Y | Y | Y | Y | Y | Y | Y | Y | Y | Y | High |
| Sun Y et al, 2016 | Sierra Leone, Low-Mid income | Ebola | Y | Y | Y | Y | Y | Y | Y | N | Y | Y | High |
| Erika Balfour et al, 2016 | United States, High-income | Various | Y | Y | Y | Y | Y | Y | Y | Y | Y | Y | High |
| R Wölfel et al, 2015 | West Africa, Low-Mid income | Ebola | Y | Y | Y | Y | Y | N | Y | Y | Y | Y | Medium |
| Randall T. Hayden et al, 2010 | United States, High-income | Influenza A H1N1 | U | Y | Y | N | N | NA | NA | N | Y | Y | Low |
| J. L. Isaac-Renton et al, 2012 | Canada, High-income | Influenza A H1N1 | Y | Y | Y | Y | Y | Y | Y | Y | Y | Y | High |
| Sheena Adamson et al, 2010 | Australia, High-income | Influenza A H1N1 | Y | Y | Y | Y | Y | Y | Y | Y | Y | Y | High |
| C. S. Sandlin et al, 2009 | United States, High-income | Chemical Terrorism | Y | Y | Y | Y | Y | Y | Y | N | Y | Y | High |
| Patricia A. Nolan et al, 2003 | United States, High-income | Bioterrorism | Y | Y | Y | Y | Y | U | Y | Y | Y | Y | Medium |

| **Category** | **Details** | **Number of Publications (%)** |
| --- | --- | --- |
| **Country Income Level** | High-income countries (HICs) | 30 |
|  | Low-and middle-income countries (LMICs) | 24 |
|  | Both income groups | 2 |
| **Study Designs** | Descriptive Study | 12 |
|  | Case Study | 37 |
|  | Cross-Sectional Study | 3 |
| **Laboratory Settings** | Public health labs | 41 |
|  | Clinical labs | 17 |
|  | Veterinary labs | 3 |
|  | Multiple laboratory types | 9 |
| **Disaster Types** | Epidemic events | 43 |
|  | Natural disaster | 6 |
|  | Human-induced disasters | 7 |
|  | Multiple laboratory types | 2 |
| **Study Quality (JBI tool)** | High | 34 (65%) |
|  | Moderate | 13 (25%) |
|  | Low | 5 (10%) |
