# Supplementary material for: The role, challenges, and solutions of laboratories in disaster medicine: a systematic review
Source: Front Public Health. 2026 Jan 13;13:1726280. doi: 10.3389/fpubh.2025.1726280 (PMC12834775; doi:10.3389/fpubh.2025.1726280)
Supplement: Supplementary file 4 [file Supplementary_file_4.docx]

**Supplementary 4: Derived from 274 primary codes grouped during the meta-aggregation process**

| **ID** | **Source (Author, Year)** | **Specific Domain** | **Primary Code (Verbatim Text)** |
| --- | --- | --- | --- |
| 1 | Adamson (2010) | T1: Infrastructure & Logistics | A large and sustained volume of community tests, especially from general practitioners in later phases, overwhelmed the laboratories. |
| 2 | Balfour (2016) | T1: Infrastructure & Logistics | A destroyed satellite laboratory during Hurricane Sandy revealed limitations; the surge in microbiology testing exceeded the Core Lab's capacity. |
| 3 | Balfour (2016) | T1: Infrastructure & Logistics | Fire (2015): Laboratory leadership was not alerted until 30 minutes after the incident, losing valuable time to activate ancillary support. |
| 4 | Clements (2024) | T1: Infrastructure & Logistics | Logistics: Difficulty in accessing appropriate equipment for efficient sample pooling. |
| 5 | Concepción-Acevedo (2018) | T1: Infrastructure & Logistics | Infrastructure: Massive damage to infrastructure, especially the island's power grid, rendered the lab system inoperable. |
| 6 | Concepción-Acevedo (2018) | T1: Infrastructure & Logistics | Logistics: Shipping companies could not pick up packages directly. A lack of dry ice and water-damaged shipping containers were also challenges. |
| 7 | de Wit (2016) | T1: Infrastructure & Logistics | Operating in a tent structure was cumbersome due to heat, rain, humidity, and wind, which affected machine performance. |
| 8 | de Wit (2016) | T1: Infrastructure & Logistics | Security: The tent structure did not provide an adequate level of security for equipment and supplies. |
| 9 | Diarra (2022) | T1: Infrastructure & Logistics | Geographic centralization: All four designated labs were located in the capital city, Bamako. |
| 10 | Feagins (2019) | T1: Infrastructure & Logistics | Poorly developed transport infrastructure and long distances often lead to prolonged transit times (sometimes weeks), degrading sample quality. |
| 11 | Feagins (2019) | T1: Infrastructure & Logistics | Equipment: Very few labs have personnel trained to maintain essential equipment like PCR machines. |
| 12 | Heller (2002) | T1: Infrastructure & Logistics | The scale of the public response and the demand for widespread environmental testing was unanticipated and threatened to overwhelm the BTRL. |
| 13 | Heller (2002) | T1: Infrastructure & Logistics | The massive surge in sample volume (3,000 times the normal level) created a huge workload, especially for environmental samples. |
| 14 | Heller (2002) | T1: Infrastructure & Logistics | The maximum throughput of the BSL-3 facility (about 40 samples per 24 hours) was the ultimate limiting factor. |
| 15 | Hendarwan (2020) | T1: Infrastructure & Logistics | Uneven distribution of labs, with 12 provinces initially having no diagnostic facility. The archipelago geography makes specimen transport difficult. |
| 16 | Isaac-Renton (2012) | T1: Infrastructure & Logistics | The pandemic caused a rapid, 10-fold surge in the number of influenza diagnostic requests. |
| 17 | Islam (2021) | T1: Infrastructure & Logistics | Infrastructure: Difficulty finding a separate space for the lab; old building architecture did not comply with ideal biosafety guidelines. |
| 18 | Islam (2021) | T1: Infrastructure & Logistics | Waste Management: Arranging a safe waste management system was a challenge. |
| 19 | Katawera (2019) | T1: Infrastructure & Logistics | The shift to enhanced surveillance caused a sudden, exponential increase in demand for lab services, overwhelming the three remaining labs. |
| 20 | Kennedy (2016) | T1: Infrastructure & Logistics | Pre-EVD, equipment frequently failed with limited repair/maintenance capacity. |
| 21 | Krishnakumari (2024) | T1: Infrastructure & Logistics | Nepal has diverse and challenging terrain, which significantly affects travel times and accessibility. |
| 22 | Mukadi-Bamuleka (2023) | T1: Infrastructure & Logistics | Chronic insecurity: Response teams were attacked by rebel groups. This led to operational disruptions, deployment delays, and breaks in the supply chain. |
| 23 | Nolan (2003) | T1: Infrastructure & Logistics | The lab and communications departments were overwhelmed with requests and concerns about strange powders, receiving 176 items in just over two weeks. |
| 24 | Rodriguez (2018) | T1: Infrastructure & Logistics | Infrastructure failure: Flooding can cause generator failure or render the entire facility inoperable, requiring evacuation. |
| 25 | Sealy (2016) | T1: Infrastructure & Logistics | International (West Africa): Weak national lab systems, unsafe specimen collection. |
| 26 | Sealy (2016) | T1: Infrastructure & Logistics | Domestic (US): Open-space, automated lab environments were not easily adapted for segregated sample management and containment. |
| 27 | Sealy (2016) | T1: Infrastructure & Logistics | Weak national laboratory systems in West Africa hindered the initial response. |
| 28 | Taylor (2014) | T1: Infrastructure & Logistics | Transport: Community transport times increased significantly (initially by 20-27 hours) due to courier stoppages, road closures, and damaged infrastructure. |
| 29 | Yacouba (2020) | T1: Infrastructure & Logistics | TAT: Before decentralization, the centralized model led to long TAT (48–72 hours), causing treatment delays and longer patient hospital stays. |
| 30 | Yu (2010) | T1: Infrastructure & Logistics | Infrastructure destruction: Communication infrastructure is often destroyed or overloaded during disasters. |
| 31 | Aden (2022) | T2: Supply Chain Instability | Initial testing capacity was limited by the availability of reagents and the requirement for manual DNA extraction. |
| 32 | Al-Waleedi (2023) | T2: Supply Chain Instability | Even the well-equipped lab in Aden sometimes ran out of reagents. |
| 33 | Bartlow (2024) | T2: Supply Chain Instability | Procurement of reagents and consumables was difficult, as some vendors have no representation in Uganda or Africa; this was exacerbated by the pandemic. |
| 34 | Diarra (2022) | T2: Supply Chain Instability | Lack of national funding: The government of Mali depended on support from partners and donors to establish needed capacity, raising sustainability questions. |
| 35 | Feagins (2019) | T2: Supply Chain Instability | Procurement: The limited number of in-country suppliers and lack of product diversity often led to stockouts of needed reagents. |
| 36 | Hayden (2010) | T2: Supply Chain Instability | Resource shortages: A quarter or more of respondents experienced shortages of collection devices, reagents, or kits. |
| 37 | Islam (2021) | T2: Supply Chain Instability | Funding: Lack of continuous funding and uncertainty about financial support. |
| 38 | Islam (2021) | T2: Supply Chain Instability | Supplies: Shortage of PPE in the initial phase due to increased global demand. |
| 39 | Kennedy (2016) | T2: Supply Chain Instability | Pre-EVD, there were frequent stockouts of reagents and consumables. |
| 40 | McLaughlin (2021) | T2: Supply Chain Instability | Supply chain shortages: High global demand caused severe shortages of essential supplies (swabs, transport media, extraction reagents). |
| 41 | Mögling (2017) | T2: Supply Chain Instability | The main challenge identified was the availability of positive control and validation materials for both molecular and serological tests. |
| 42 | Reusken (2020) | T2: Supply Chain Instability | The top challenge was the initial lack of positive control materials, followed by a lack of primers and/or probes. |
| 43 | Sealy (2016) | T2: Supply Chain Instability | Domestic (US): Manufacturers refused to warranty equipment used for Ebola testing, leading to liability concerns and some labs refusing samples. |
| 44 | Sealy (2016) | T2: Supply Chain Instability | International (West Africa): Inadequate supply chain. |
| 45 | Stanislawski (2023) | T2: Supply Chain Instability | Shortages of consumables and safety equipment were a major challenge, especially early in the pandemic. |
| 46 | Toney (2021) | T2: Supply Chain Instability | Severe shortages of multiple testing components (swabs, PPE, reagents) due to global demand and supply chain disruptions. |
| 47 | Yacouba (2020) | T2: Supply Chain Instability | Shortages of RNA extraction kits occurred, leading to prioritization of testing for vulnerable groups. |
| 48 | Adamson (2010) | T3: Workforce Constraints | A concurrent whooping cough outbreak adversely affected the capacity of some labs, as they had to use shared equipment and staff for other urgent tests. |
| 49 | Clements (2024) | T3: Workforce Constraints | Personnel: Limited staff capacity and difficulties in managing work schedules to prevent burnout. |
| 50 | Crawford (2010) | T3: Workforce Constraints | The high level of expertise required for virology and molecular testing meant staff could not be augmented in the short term, stressing the existing team. |
| 51 | Crawford (2010) | T3: Workforce Constraints | Staff dedication and unwillingness to take time off created a risk of burnout, which management had to address with mandatory breaks. |
| 52 | de Wit (2016) | T3: Workforce Constraints | The low throughput of sample processing in the biosafety cabinet sometimes caused delays. |
| 53 | Donnelly (2023) | T3: Workforce Constraints | Testing was performed by clinical staff with no prior formal training in laboratory practices or quality assurance procedures. |
| 54 | Feagins (2019) | T3: Workforce Constraints | A shortage of skilled laboratory professionals exists in sub-Saharan Africa. Recruiting and retaining skilled staff in public labs is difficult due to poor pay. |
| 55 | Grigoryan (2025) | T3: Workforce Constraints | In the initial phase, there was a lack of staff, a large workload, fear of the new virus, and fatigue. |
| 56 | Hayden (2010) | T3: Workforce Constraints | Personnel shortages: 16.1% of facilities experienced personnel shortages due to testing volume. |
| 57 | Islam (2021) | T3: Workforce Constraints | Personnel: Difficulty motivating staff, insufficient staff numbers leading to burnout; lack of risk allowance. |
| 58 | Jean Louis (2017) | T3: Workforce Constraints | High staff turnover rates affect the quality of testing. |
| 59 | Jeon (2023) | T3: Workforce Constraints | Despite adding personnel, work fatigue was high, requiring staff rotation. |
| 60 | Katawera (2019) | T3: Workforce Constraints | Initial efforts to recruit more international experts were unsuccessful due to lengthy recruitment procedures. |
| 61 | Mögling (2017) | T3: Workforce Constraints | Personnel capacity was a challenge for many. The high price of commercial assays was a barrier for 15 labs. |
| 62 | Reusken (2020) | T3: Workforce Constraints | Lack of personnel/time was the second most-cited challenge to implementation. |
| 63 | Rodriguez (2018) | T3: Workforce Constraints | On-site "ride-out" teams face significant risks of fatigue, illness, and injury during prolonged events. |
| 64 | Rodriguez (2018) | T3: Workforce Constraints | Relief staff are often unable to report for duty due to transportation barriers and personal property damage post-disaster. |
| 65 | Safadel (2024) | T3: Workforce Constraints | The sustainability of the workforce is a serious challenge. Strategies to motivate staff (e.g., competitive pay) were not uniformly implemented. |
| 66 | Stanislawski (2023) | T3: Workforce Constraints | The safe deployment of tests was limited to trained personnel, which could be difficult in some areas. High personnel demand to operate each station. |
| 67 | Sun (2016) | T3: Workforce Constraints | Limited local capacity: Before the team's arrival, only three international labs existed, and their combined capacity could not meet the demand. |
| 68 | Yacouba (2020) | T3: Workforce Constraints | Work overload: Technicians were frequently overloaded due to high sample volumes and manual RNA extraction. |
| 69 | Yacouba (2020) | T3: Workforce Constraints | Manual extraction: Manual RNA extraction was time-consuming and had a high risk of cross-contamination. |
| 70 | Yu (2010) | T3: Workforce Constraints | Training: Deploying complex connectivity systems in austere environments is difficult. Language and education barriers can be significant obstacles. |
| 71 | Adamson (2010) | T4: Quality, biosafety, and chain of custody | The high volume of community tests negatively impacted the provision of timely results for priority cases (e.g., hospitalized patients). |
| 72 | Al-Waleedi (2023) | T4: Quality, biosafety, and chain of custody | Data Quality: Low reporting rates and questionable data quality. Data collection is seen as a work burden, especially without electronic records or internet. |
| 73 | de Wit (2016) | T4: Quality, biosafety, and chain of custody | Case management pressure: A large number of patients and a shortage of beds in ETUs during the peak forced the adoption of a less stringent testing algorithm for patient discharge. |
| 74 | Diarra (2022) | T4: Quality, biosafety, and chain of custody | Initial limited testing capacity meant clinicians had to rely on imaging (X-ray and CT) for diagnosis. |
| 75 | Donnelly (2023) | T4: Quality, biosafety, and chain of custody | Technical issues: Risk of false-positive results (flagged by FDA) required high vigilance, re-testing, and investigation, increasing workload. |
| 76 | Grigoryan (2025) | T4: Quality, biosafety, and chain of custody | Gaps existed between current biosafety management and quality control practices and appropriate practices. Internal quality control was not systematically enforced or documented. |
| 77 | Hayden (2010) | T4: Quality, biosafety, and chain of custody | A significant portion of labs relied on rapid antigen tests, which have limited sensitivity and specificity for H1N1. |
| 78 | Hayden (2010) | T4: Quality, biosafety, and chain of custody | Delays in confirmatory testing: Nearly half (45.1%) of respondents reported delays in confirmatory subtyping from public health labs. |
| 79 | Heller (2002) | T4: Quality, biosafety, and chain of custody | The original BTRL was contaminated with B. anthracis spores during the sampling of the first positive letter, exposing three staff members and forcing its closure. |
| 80 | Mérens (2012) | T4: Quality, biosafety, and chain of custody | The DNA extraction step showed large variations in performance, with a maximum difference of 12.72 Ct between labs for the same sample. |
| 81 | Mérens (2012) | T4: Quality, biosafety, and chain of custody | Result interpretation: A lack of or inappropriate interpretation often accompanied results; results must be clearly explained for non-specialists to make decisions. |
| 82 | Mögling (2017) | T4: Quality, biosafety, and chain of custody | Quality/Certification: 5 of the diagnosing labs had no ISO accreditation at all. |
| 83 | Nolan (2003) | T4: Quality, biosafety, and chain of custody | Initial screening procedures for environmental samples were not stringent enough and did not rule out enough non-credible materials (e.g., laundry powder samples, junk mail). |
| 84 | Reusken (2020) | T4: Quality, biosafety, and chain of custody | Lack of panels to assess test specificity was a significant barrier. Proper validation of specificity was lacking in the majority of labs that had implemented testing. |
| 85 | Sealy (2016) | T4: Chain-of-custody and biosafety | Unsafe specimen collection and transport practices were identified as a major biosafety challenge. |
| 86 | Toney (2021) | T4: Quality, biosafety, and chain of custody | The initial CDC test kits were unreliable, and bureaucratic hurdles delayed the development of alternative tests. |
| 87 | Venturi (2020) | T4: Quality, biosafety, and chain of custody | Lack of standardization across Europe in procedures, case definitions, and clinician testing behavior biased data collection. |
| 88 | Villanueva (2019) | T4: Quality, biosafety, and chain of custody | During the Ebola outbreak, "significant gaps in biosafety and biosecurity practices" were identified. |
| 89 | Aden (2022) | T5: Administrative and regulatory barriers | Some clinicians and patients reported difficulties navigating public health testing procedures, including obtaining testing authorization. |
| 90 | Al-Waleedi (2023) | T5: Administrative and regulatory barriers | Coordination: The flow of information between administrative levels was a "major obstacle" and inconsistent. No established network between MOPH&P and the veterinary sector. |
| 91 | Bacchus (2021) | T5: Administrative and regulatory barriers | Lack of awareness: Civil health authorities were not always aware of the unique capabilities of specialized military units. |
| 92 | Bacchus (2021) | T5: Administrative and regulatory barriers | Interagency cooperation was often based on personal relationships rather than institutionalized formal plans, risking continuity. |
| 93 | Clements (2024) | T5: Administrative and regulatory barriers | The greatest barrier: obtaining CLIA certification was a significant obstacle. |
| 94 | Clements (2024) | T5: Administrative and regulatory barriers | Leadership: Resistance from the leadership of some organizations due to concerns about costs or negative impacts on student enrollment. |
| 95 | Clements (2024) | T5: Administrative and regulatory barriers | Collaboration: Lack of support or encountering barriers from human health agencies. |
| 96 | Diao (2024) | T5: Administrative and regulatory barriers | A tendency for overprotection was identified, which wastes resources and can reduce efficiency. |
| 97 | Donnelly (2023) | T5: Administrative and regulatory barriers | Demand Management: Inappropriate use of limited POCT resources was a challenge. Staff from outside the ED attempted to circumvent pathways to get faster results. |
| 98 | Grigoryan (2025) | T5: Administrative and regulatory barriers | The coordination of the testing network and the entire laboratory system was suboptimal. |
| 99 | Islam (2021) | T5: Administrative and regulatory barriers | Accreditation: Could not obtain accreditation for the lab due to the ongoing pandemic. |
| 100 | Jeon (2023) | T5: Administrative and regulatory barriers | Automation of the pooling process faced challenges due to the diversity of sample container sizes in Korea. |
| 101 | Nolan (2003) | T5: Administrative and regulatory barriers | The response was primarily environmental, with law enforcement as the main partner, whereas preparedness had focused more on partnerships with clinicians for human cases. |
| 102 | Salerno (2020) | T5: Administrative and regulatory barriers | CLIA regulations for verifying an EUA test were not specific, leading to cautious and varied implementation. |
| 103 | Sealy (2016) | T5: Administrative and regulatory barriers | Domestic (US): Manufacturers refused to warranty equipment used for Ebola testing, leading to liability concerns and some labs refusing samples. |
| 104 | Stanislawski (2023) | T5: Administrative and regulatory barriers | Adjusting legal frameworks (e.g., disinfectant production or transport regulations) was necessary to enable operations under pandemic conditions. |
| 105 | Toney (2021) | T5: Administrative and regulatory barriers | Loosened regulations for some test types, challenges with data reporting, and an erosion of public trust due to political influence. |
| 106 | Williams (2014) | T5: Administrative and regulatory barriers | Lack of Preparedness: Inertia or opposition to planning can be a significant obstacle. Lab plans are not integrated with the larger hospital/facility plans. |
| 107 | Abu-Dayyeh (2023) | T6: IT & Data Interoperability | Initial challenges included the lack of a dedicated IT solution for communicating with patients at high throughput. |
| 108 | Adamson (2010) | T6: IT & Data Interoperability | Data entry and reporting into NetEpi were done manually and were labor-intensive. |
| 109 | Balfour (2016) | T6: IT & Data Interoperability | Different LIS systems were a major barrier to redirecting test volume after a lab was destroyed. |
| 110 | Bartlow (2024) | T6: IT & Data Interoperability | Unstable internet connectivity affects communication and the use of web-based analysis tools. |
| 111 | Diarra (2022) | T6: IT & Data Interoperability | Returning results to patients was sometimes impossible due to incorrect contact information or patient movement. |
| 112 | Donnelly (2023) | T6: IT & Data Interoperability | A major challenge was that the hospital's IT infrastructure did not natively support POCT results, requiring a cumbersome manual verification process by lab staff. |
| 113 | Hayden (2010) | T6: IT & Data Interoperability | Information confusion: Over 40% reported some confusion between media messages and information from authoritative sources. |
| 114 | Heller (2002) | T6: IT & Data Interoperability | A viable database system had to be built hastily to track thousands of samples. |
| 115 | Hendarwan (2020) | T6: IT & Data Interoperability | Personnel constraints (both in number and skills) were a major difficulty. |
| 116 | McLaughlin (2021) | T6: IT & Data Interoperability | The initial manual intake system was overwhelmed by high sample volumes. Submitted specimens often lacked required information. |
| 117 | Mukadi-Bamuleka (2023) | T6: IT & Data Interoperability | Any issues with spelling or transcription in data had to be resolved manually by using other patient identifiers. |
| 118 | Rodriguez (2018) | T6: IT & Data Interoperability | Communication: Failure of phone or IT systems can disrupt communication, test ordering (CPOE), and result reporting (LIS). |
| 119 | Salerno (2020) | T6: IT & Data Interoperability | Different states had different reporting requirements, posing a challenge for national commercial labs. |
| 120 | Sandlin (2009) | T6: IT & Data Interoperability | The diversity of vendor-based operating systems for different analytical instruments is a significant challenge when designing and implementing a LIMS. |
| 121 | Sandlin (2009) | T6: IT & Data Interoperability | Labs generate a massive amount of analytical data that must be processed efficiently for review, especially in an emergency. |
| 122 | Sealy (2016) | T6: IT & Data Interoperability | International (West Africa): Incomplete documentation (symptom onset, patient info) hindered data linkage and follow-up. |
| 123 | Venturi (2020) | T6: IT & Data Interoperability | Routine LIMS in most labs are not set up to allow easy data extraction for such queries. |
| 124 | Venturi (2020) | T6: IT & Data Interoperability | Epidemiological parameters, like travel destination, are often not provided with the diagnostic request, leading to incomplete reporting. |
| 125 | Villanueva (2019) | T6: IT & Data Interoperability | During the Zika outbreak, the massive testing volume created a "bottleneck in data entry," hindering timely reporting of results. |
| 126 | Yu (2010) | T6: IT & Data Interoperability | Wireless networks are vulnerable to unauthorized access and risk violating patient privacy rules (e.g., HIPAA). |
| 127 | Heller (2002) | T7: Cybersecurity vulnerabilities | Regular third-party system vulnerability assessments and penetration testing are conducted. |
| 128 | Kennedy (2016) | T7: Cybersecurity vulnerabilities | Secure, encrypted communication channels are established as the sole method for transmitting results. |
| 129 | Sandlin (2009) | T7: Cybersecurity vulnerabilities | Mandatory, recurring cybersecurity training for all laboratory staff is implemented. |
| 130 | Sandlin (2009) | T7: Cybersecurity vulnerabilities | Multi-factor authentication is implemented for all systems containing sensitive patient or laboratory data. |
| 131 | Sandlin (2009) | T7: Cybersecurity vulnerabilities | Site-to-site Virtual Private Networks (VPNs) are used for secure data transfer between network laboratories. |
| 132 | Toney (2021) | T7: Cybersecurity vulnerabilities | A formal incident response plan for cyber attacks is developed and tested. |
| 133 | Williams (2014) | T7: Cybersecurity vulnerabilities | Always prepare for communication failure by having redundant systems and processes (e.g., amateur radio, satellite phones). |
| 134 | Williams (2014) | T7: Cybersecurity vulnerabilities | Have backup LIS and HIS systems, or well-planned manual procedures (e.g., logbooks), for when the primary system fails. |
| 135 | Williams (2014) | T7: Cybersecurity vulnerabilities | Communication failure is considered the "greatest challenge" in large-scale disaster operations; phone systems are very likely to be inoperable or overloaded. |
| 136 | Yu (2010) | T7: Cybersecurity vulnerabilities | Disaster planning needs to account for the deployment of secure wireless networks (e.g., using WPA/WPA2 encryption). |
| **Theme 3: Solutions** | | | |
| 137 | Abu-Dayyeh (2023) | G1: Redundant infrastructure and mobile deployments | Established 30 collection centers and 6 adjoining PCR labs at various locations, including drive-thru, walk-in, and border-crossing sites. |
| 138 | Adamson (2010) | G1: Redundant infrastructure and mobile deployments | Lab scale-up: Initially, nucleic acid testing (NAT) was only performed at two public reference labs. As test numbers increased, six other public labs joined the response. |
| 139 | Aden (2022) | G1: Redundant infrastructure and mobile deployments | Expanded testing to five large commercial laboratories to increase access and convenience for providers and patients. |
| 140 | Bacchus (2021) | G1: Redundant infrastructure and mobile deployments | The mobile lab (B-FAL) is a self-sufficient, container-based unit with a Class III biosafety cabinet (glovebox). |
| 141 | Balfour (2016) | G1: Redundant infrastructure and mobile deployments | The integrated lab service acted as a regional surge resource, absorbing a 30% test volume increase with only 24 hours' notice. |
| 142 | Clements (2024) | G1: Redundant infrastructure and mobile deployments | Existing Biosafety Level 2 and 3 facilities are available to handle diseases of concern. |
| 143 | Crawford (2010) | G1: Redundant infrastructure and mobile deployments | Facilities: Completed an immediate expansion of physical facilities, creating a supplementary negative-pressure lab space within 4 days. |
| 144 | de Wit (2016) | G1: Redundant infrastructure and mobile deployments | The field lab demonstrated high throughput, processing nearly 6,000 samples over a 9-month period. |
| 145 | de Wit (2016) | G1: Redundant infrastructure and mobile deployments | Surge capacity was proven by processing over 350 samples per week during the peak of the outbreak. |
| 146 | Feagins (2019) | G1: Redundant infrastructure and mobile deployments | Organized training courses on specimen referral networks and handling. Piloted a transport system using the national postal service. |
| 147 | Gehre (2024) | G1: Redundant infrastructure and mobile deployments | Deployed mobile labs to local outbreak sites can dramatically shorten diagnostic turnaround times and solve logistical challenges. |
| 148 | Heller (2002) | G1: Redundant infrastructure and mobile deployments | A Bio-Terrorism Response Lab Command Center was established to direct and coordinate all activities. |
| 149 | Isaac-Renton (2012) | G1: Redundant infrastructure and mobile deployments | The workflow was redesigned from a single-technician model to a "flow cell" model, where each staff member was responsible for a specific task. |
| 150 | Islam (2021) | G1: Redundant infrastructure and mobile deployments | Collaborative efforts, local funding, and in-country expertise can enable the establishment of a biocontainment facility in a short time. |
| 151 | Katawera (2019) | G1: Redundant infrastructure and mobile deployments | Established a new mobile laboratory (ELWA III) and a new testing point at a clinical lab (Redemption Hospital). |
| 152 | Kennedy (2016) | G1: Redundant infrastructure and mobile deployments | A sophisticated and well-coordinated sample referral system supported by Riders for Health was established to ensure timely sample transport. |
| 153 | Mérens (2012) | G1: Redundant infrastructure and mobile deployments | The network utilizes a three-level structure for response (Level 1: Sampling, Level 2: Screening, Level 3: Confirmation). |
| 154 | Mérens (2012) | G1: Redundant infrastructure and mobile deployments | Level 2 screening is conducted by a diverse group of university, military, environmental, and veterinary laboratories. |
| 155 | Nolan (2003) | G1: Redundant infrastructure and mobile deployments | A remote screening location was established to examine all items for incendiary devices, radiation, and volatile organic compounds before they reached the lab. |
| 156 | Rodriguez (2018) | G1: Redundant infrastructure and mobile deployments | Convincing administration to move laboratories to higher floors is essential for successful hospital function against hurricane threats. |
| 157 | Sealy (2016) | G1: Redundant infrastructure and mobile deployments | In the US: Years of preparation and coordinated activities created robust and adaptable lab systems. The existing LRN was a valuable asset. |
| 158 | Stanislawski (2023) | G1: Redundant infrastructure and mobile deployments | Established a mobile, container-based screening facility with an integrated BSL-2 lab within 4 weeks of the first lockdown in Germany. |
| 159 | Sun (2016) | G1: Redundant infrastructure and mobile deployments | A conventional laboratory space was converted into a temporary BSL-II laboratory with 3 working areas and 2 buffer zones within 6 days. |
| 160 | Taylor (2014) | G1: Redundant infrastructure and mobile deployments | Existing automated biochemistry analyzers were upgraded to manage surge sample loads. |
| 161 | Taylor (2014) | G1: Redundant infrastructure and mobile deployments | A process review led to an expanded registration area and training all new staff on a new system based on Lean principles. |
| 162 | Villanueva (2019) | G1: Redundant infrastructure and mobile deployments | The LRN's strength comes from its diverse composition, including public health, veterinary, military, and food testing facilities. |
| 163 | Villanueva (2019) | G1: Redundant infrastructure and mobile deployments | The network is comprised of 123 domestic reference laboratories in a tiered system. |
| 164 | Williams (2014) | G1: Redundant infrastructure and mobile deployments | Mortuary Plan: The hospital needs a mass fatality management plan, coordinated with the community plan, in which the lab has a role. |
| 165 | Wölfel (2015) | G1: Redundant infrastructure and mobile deployments | The entire mobile laboratory is packed in wheeled, hard-shell boxes, transportable as regular luggage on commercial airlines. |
| 166 | Yacouba (2020) | G1: Redundant infrastructure and mobile deployments | Decentralized rRT-PCR testing to three other regions. This significantly reduced TAT from 48-72 hours to 12-24 hours. |
| 167 | Yang (2017) | G1: Redundant infrastructure and mobile deployments | A well-established national laboratory system is coordinated by the TCDC. |
| 168 | Yang (2017) | G1: Redundant infrastructure and mobile deployments | Operates a tightly connected specimen referral network, adhering to standard regulations. |
| 169 | Adamson (2010) | G2: Diversified sourcing and stockpiling strategies | After the outbreak, most facilities planned to increase resources for the next flu season, primarily by increasing stockpiles of reagents/kits (84.6%). |
| 170 | Bacchus (2021) | G2: Diversified sourcing and stockpiling strategies | The mobile lab (B-FAL) was pre-stocked with reagents and supplies in autumn 2019, which was crucial due to subsequent global shortages. |
| 171 | Bacchus (2021) | G2: Diversified sourcing and stockpiling strategies | Continuous pre-stocking of reagents and supplies during non-crisis periods is essential to avoid being limited by supply chain disruptions. |
| 172 | Feagins (2019) | G2: Diversified sourcing and stockpiling strategies | Established the CDC's International Reagent Resource (IRR) program for GHSA countries to procure reagents free of charge. |
| 173 | Feagins (2019) | G2: Diversified sourcing and stockpiling strategies | Piloted procurement systems through vendors in Africa, France, and the US to find efficient solutions. |
| 174 | Gehre (2023) | G2: Diversified sourcing and stockpiling strategies | Supply chains are strengthened by diversifying suppliers at a regional level to reduce dependency on single sources. |
| 175 | Islam (2021) | G2: Diversified sourcing and stockpiling strategies | Local funding and in-country expertise can enable the establishment of a biocontainment facility. (Implicitly enabling local sourcing). |
| 176 | McLaughlin (2021) | G2: Diversified sourcing and stockpiling strategies | Proactively ordered supplies and reagents to achieve maximum surge testing capacity. |
| 177 | McLaughlin (2021) | G2: Diversified sourcing and stockpiling strategies | Identified alternative sources for media and reagents. |
| 178 | Reusken (2020) | G2: Diversified sourcing and stockpiling strategies | Positive control materials and panels for specificity testing were made available early via the European Virus Archive (EVAg). |
| 179 | Safadel (2024) | G2: Diversified sourcing and stockpiling strategies | Promoted and supported domestic manufacturers to produce PCR/antigen test kits, PPE, and safety equipment. |
| 180 | Safadel (2024) | G2: Diversified sourcing and stockpiling strategies | Managed the centralized procurement and distribution of in-vitro diagnostics (IVDs) for the public sector. |
| 181 | Stanislawski (2023) | G2: Diversified sourcing and stockpiling strategies | Overcame supply shortages by self-producing custom, pre-labeled test kits and disinfectants. |
| 182 | Sun (2016) | G2: Diversified sourcing and stockpiling strategies | More than 1,000 types of medical and logistical supplies (108 tons total) were packed and transported based on projected local needs. |
| 183 | Toney (2021) | G2: Diversified sourcing and stockpiling strategies | Used multiple types of tests from different manufacturers to address supply chain shortages. |
| 184 | Toney (2021) | G2: Diversified sourcing and stockpiling strategies | Recommendation: Stockpile supplies for future pandemics, especially swabs, PPE, and other non-perishable items. |
| 185 | Toney (2021) | G2: Diversified sourcing and stockpiling strategies | Recommendation: The National Strategic Stockpile should contain testing materials like collection kits and viral transport media. |
| 186 | Toney (2021) | G2: Diversified sourcing and stockpiling strategies | Recommendation: A nationally coordinated inventory system and a strategic stockpile of testing materials are needed. |
| 187 | Williams (2014) | G2: Diversified sourcing and stockpiling strategies | Increase inventory, arrange contracts with vendors to ensure rapid resupply, and establish Memorandums of Understanding (MOUs) with other labs to share/borrow reagents. |
| 188 | Yacouba (2020) | G2: Diversified sourcing and stockpiling strategies | Diversified rRT-PCR kits to minimize the risk of shortages and increase the availability of reagents. |
| 189 | Abu-Dayyeh (2023) | G3: Workforce surge mechanisms and cross-training programs | Staff support included financial incentives and increased paid leave to maintain morale and engagement. |
| 190 | Abu-Dayyeh (2023) | G3: Workforce surge mechanisms and cross-training programs | On-site health resources, such as small clinics and dedicated mental health support, were provided for staff. |
| 191 | Abu-Dayyeh (2023) | G3: Workforce surge mechanisms and cross-training programs | Implemented wellness and mental health support for staff, including financial incentives, mental health support, and increased paid leave. |
| 192 | Balfour (2016) | G3: Workforce surge mechanisms and cross-training programs | The most critical strength was the pre-existing culture of support and working relationships among the 2000+ staff, which enabled an immediate, coordinated response. |
| 193 | Clements (2024) | G3: Workforce surge mechanisms and cross-training programs | Veterinary laboratories already possess experience in quality assurance, biosafety, and high-throughput testing. |
| 194 | Crawford (2010) | G3: Workforce surge mechanisms and cross-training programs | Workforce: Augmented the lab workforce by deploying licensed support and research staff. Extended work hours significantly. |
| 195 | Gehre (2023) | G3: Workforce surge mechanisms and cross-training programs | A "Training of Trainers" program was used to build upon existing capacity for mpox diagnostic training. |
| 196 | Grigoryan (2025) | G3: Workforce surge mechanisms and cross-training programs | Provided training on PCR testing and Infection Prevention and Control (IPC) measures for staff. |
| 197 | Heller (2002) | G3: Workforce surge mechanisms and cross-training programs | Re-assigned staff from other units and requested support from a Department of Defense (DOD) Microbiology Rapid Response Team. |
| 198 | Isaac-Renton (2012) | G3: Workforce surge mechanisms and cross-training programs | A multidisciplinary Lean team was established to apply Lean methods to the influenza workflow. |
| 199 | Isaac-Renton (2012) | G3: Workforce surge mechanisms and cross-training programs | Staffing changes: Cross-trained medical lab technologists from other departments to staff the new flow cells. |
| 200 | Islam (2021) | G3: Workforce surge mechanisms and cross-training programs | Staff were trained on-site in basic techniques, equipment operation, and IPC practices before starting. |
| 201 | Islam (2021) | G3: Workforce surge mechanisms and cross-training programs | The department had long-standing experience in molecular detection of blood-borne pathogens, providing a foundation for the team. |
| 202 | Katawera (2019) | G3: Workforce surge mechanisms and cross-training programs | Recruited and trained 16 new technicians in EVD molecular diagnostics and implemented part-time shifts to extend testing hours. |
| 203 | Mérens (2012) | G3: Workforce surge mechanisms and cross-training programs | The exercises were a key preparedness activity to test essential points and train staff. |
| 204 | Nolan (2003) | G3: Workforce surge mechanisms and cross-training programs | Lesson Learned: A "no-notice drill" (a real event) is a challenging but realistic and valuable teaching tool. |
| 205 | Rodriguez (2018) | G3: Workforce surge mechanisms and cross-training programs | Mitigate staff fatigue by providing appropriate lodging and mental health support (e.g., disaster behavioral health teams). |
| 206 | Safadel (2024) | G3: Workforce surge mechanisms and cross-training programs | Developed and deployed online training programs and workshops on quality and safety. |
| 207 | Sun (2016) | G3: Workforce surge mechanisms and cross-training programs | The team received a 3-week training course in Beijing before departure, focusing on multi-skilling each member. |
| 208 | Taylor (2014) | G3: Workforce surge mechanisms and cross-training programs | Partnered with one of the destroyed labs to take on their staff to help manage the increased workload. |
| 209 | Williams (2014) | G3: Workforce surge mechanisms and cross-training programs | Personnel: Build a notification system (e.g., call tree), and plan for lodging, transportation, and child/pet care for staff. |
| 210 | Yacouba (2020) | G3: Workforce surge mechanisms and cross-training programs | Mobilized trained PCR technicians from other centers to support the central laboratory. |
| 211 | Abu-Dayyeh (2023) | G4: Adoption of EQA schemes and harmonized SOPs | Partnership success was facilitated by unifying diagnostic methodologies, such as all partners using the same PCR kits. |
| 212 | Adamson (2010) | G4: Adoption of EQA schemes and harmonized SOPs | Test development: Reference labs rapidly developed new NAT assays and evaluated them with WHO control materials. |
| 213 | Balfour (2016) | G4: Adoption of EQA schemes and harmonized SOPs | A system-wide, unified quality management program ensures continuous inspection readiness across all labs. |
| 214 | Crawford (2010) | G4: Adoption of EQA schemes and harmonized SOPs | Testing Model: Switched from routine screening tests (rapid antigen) and viral culture to a high-capacity molecular test (RVP). |
| 215 | de Wit (2016) | G4: Adoption of EQA schemes and harmonized SOPs | The sample inactivation efficiency of the RNA extraction kit was evaluated prior to the field mission to establish a safe workflow. |
| 216 | Grigoryan (2025) | G4: Adoption of EQA schemes and harmonized SOPs | Deployed a national External Quality Assessment (EQA) program for COVID-19 PCR testing, led by the NCDC. |
| 217 | Heller (2002) | G4: Adoption of EQA schemes and harmonized SOPs | Upgraded all environmental sample testing to strict BSL-3 containment protocols and areas. |
| 218 | Islam (2021) | G4: Adoption of EQA schemes and harmonized SOPs | Standard Operating Procedures (SOPs) were developed and followed for SARS-CoV-2 RNA detection. |
| 219 | Mérens (2012) | G4: Adoption of EQA schemes and harmonized SOPs | A key to successful detection is staff training and the standardization of methods deployed by all labs in the network. |
| 220 | Sandlin (2009) | G4: Adoption of EQA schemes and harmonized SOPs | Automated quality assurance reviews and notifications were used to allow statisticians and supervisors to review and approve from remote locations. |
| 221 | Sun (2016) | G4: Adoption of EQA schemes and harmonized SOPs | Passed a single-blind test with 5 samples from a WHO network lab with 100% accuracy, using self-developed detection kits. |
| 222 | Villanueva (2019) | G4: Adoption of EQA schemes and harmonized SOPs | To address biosafety challenges from the Ebola outbreak, the CDC/APHL Biosafety and Biosecurity Program was established to enhance practices through training and support. |
| 223 | Williams (2014) | G4: Adoption of EQA schemes and harmonized SOPs | Forensics: Ensure security and protection of potential evidence related to criminal or terrorist activity through a chain-of-custody (COC) process. |
| 224 | Wölfel (2015) | G4: Adoption of EQA schemes and harmonized SOPs | The technical concept of the EMLab became a blueprint for other Ebola mobile labs established in West Africa. |
| 225 | Yang (2017) | G4: Adoption of EQA schemes and harmonized SOPs | Designated, contract, and licensed labs must undergo periodic document reviews, on-site inspections, or proficiency testing to ensure diagnostic quality. |
| 226 | Adamson (2010) | G5: Proactive regulatory navigation and platform standardization | The response was guided by pre-existing national and NSW pandemic influenza plans which defined lab operations based on pandemic phases. |
| 227 | Bacchus (2021) | G5: Proactive regulatory navigation and platform standardization | Pre-existing civil-military exercises (in 2018 and 2019) were conducted to harmonize methods and establish communication channels. |
| 228 | Balfour (2016) | G5: Proactive regulatory navigation and platform standardization | Standardization of operations and technology was achieved through the creation of 16 cross-functional "common standard committees". |
| 229 | Bartlow (2024) | G5: Proactive regulatory navigation and platform standardization | Building trust is an essential and long-term element of successful collaboration. |
| 230 | Clements (2024) | G5: Proactive regulatory navigation and platform standardization | Urgently need to reduce legal barriers (especially CLIA regulations) to allow veterinary labs to participate quickly. |
| 231 | Clements (2024) | G5: Proactive regulatory navigation and platform standardization | Policymakers and regulatory agencies need to recognize the contributions of the veterinary community and be willing to collaborate in a One Health approach. |
| 232 | Donnelly (2023) | G5: Proactive regulatory navigation and platform standardization | Governance is key: Establishing clear governance under laboratory direction, defining roles, and ensuring open communication is key to success. |
| 233 | Hayden (2010) | G5: Proactive regulatory navigation and platform standardization | The outbreak highlighted resource shortages (supplies, staff), providing valuable lessons for future preparedness. |
| 234 | Heller (2002) | G5: Proactive regulatory navigation and platform standardization | Implemented a triage algorithm in collaboration with the FBI to prioritize the testing of thousands of suspicious items. |
| 235 | Hendarwan (2020) | G5: Proactive regulatory navigation and platform standardization | A strong registration and reporting system is needed to integrate data from various sources. |
| 236 | Islam (2021) | G5: Proactive regulatory navigation and platform standardization | Relevant guidelines from CDC and WHO were consulted during the planning phase. |
| 237 | Krishnakumari (2024) | G5: Proactive regulatory navigation and platform standardization | The use of publicly available data and mathematical optimization should be considered for future planning. |
| 238 | Mahmoud (2022) | G5: Proactive regulatory navigation and platform standardization | A "One Health knowledge and epidemic commission" should be established to engage all relevant stakeholders. |
| 239 | Mögling (2017) | G5: Proactive regulatory navigation and platform standardization | A national and/or EU contingency fund should be established to ensure adequate and robust laboratory preparedness and response. |
| 240 | Nolan (2003) | G5: Proactive regulatory navigation and platform standardization | An incident command system was activated by the Emergency Management Agency (EMA), bringing together the FBI, HEALTH, State Fire Marshal, HAZMAT, and National Guard. |
| 241 | Nolan (2003) | G5: Proactive regulatory navigation and platform standardization | A triage plan for environmental samples was established in collaboration with the FBI to prioritize items for processing. |
| 242 | Pavlin (2000) | G5: Proactive regulatory navigation and platform standardization | The concept of a national infectious disease laboratory network is a goal that must be achieved for preparedness. |
| 243 | Sealy (2016) | G5: Proactive regulatory navigation and platform standardization | Establishment of Emergency Operations Centers (EOCs) was a key component of the response coordination structure. |
| 244 | Sealy (2016) | G5: Proactive regulatory navigation and platform standardization | EOCs provided labs with a formal mechanism for receiving feedback, enabling rapid process improvement. |
| 245 | Sealy (2016) | G5: Proactive regulatory navigation and platform standardization | Better mechanisms are needed to streamline the development and evaluation of new diagnostic tests. |
| 246 | Sealy (2016) | G5: Proactive regulatory navigation and platform standardization | In the US, a continued review and refinement of laboratory biosafety practices is needed. |
| 247 | Williams (2014) | G5: Proactive regulatory navigation and platform standardization | Planning & Integration: All labs must have an Emergency Operations Plan (EOP), and this plan must be integrated with institutional and community plans. |
| 248 | Williams (2014) | G5: Proactive regulatory navigation and platform standardization | Legal & Ethical: Consult legal experts regarding labor laws and potential liabilities during emergency operations. |
| 249 | Williams (2014) | G5: Proactive regulatory navigation and platform standardization | All labs must have an Emergency Operations Plan (EOP), and this plan must be integrated with institutional and community plans. |
| 250 | Yang (2017) | G5: Proactive regulatory navigation and platform standardization | Encouraged a close collaborative relationship between public health and veterinary laboratories to achieve "One Health" goals. |
| 251 | Abu-Dayyeh (2023) | G6: Middleware integration and centralized LIMS | Introduced an end-to-end digital IT platform to manage samples, registration, result dissemination, and reporting to the MoH. |
| 252 | Abu-Dayyeh (2023) | G6: Middleware integration and centralized LIMS | Real-time linkage of the private laboratory's results to the Ministry of Health's server was a key technical enabler for the partnership. |
| 253 | Adamson (2010) | G6: Middleware integration and centralized LIMS | The planned response was based on the lab's Pandemic Influenza Management System (PIMS) and a web-based disease management tool, NetEpi, as the single database. |
| 254 | Balfour (2016) | G6: Middleware integration and centralized LIMS | An integrated Laboratory Information System (LIS) across most labs was an essential component of the response. |
| 255 | Concepción-Acevedo (2018) | G6: Middleware integration and centralized LIMS | An alternative reporting method using a secure File Transfer Protocol (FTP) site was established for data exchange. |
| 256 | Crawford (2010) | G6: Middleware integration and centralized LIMS | Information Systems: Built an interface for the Laboratory Information System (LIS) to handle new testing and billing across multiple hospital systems. |
| 257 | Grigoryan (2025) | G6: Middleware integration and centralized LIMS | A single national electronic health data management system (Armed) was successfully deployed for COVID-19. |
| 258 | Heller (2002) | G6: Middleware integration and centralized LIMS | Developed a new database to track samples and results. |
| 259 | Nolan (2003) | G6: Middleware integration and centralized LIMS | The communications team handled all media contact and public information calls to save time for laboratory personnel. |
| 260 | Sandlin (2009) | G6: Middleware integration and centralized LIMS | The primary solution was a custom-built Laboratory Information Management System (LIMS), called the Emergency Response Management System (ERMS). |
| 261 | Sandlin (2009) | G6: Middleware integration and centralized LIMS | The system was designed to be scalable, allowing for the rapid addition of new instruments to meet surge capacity needs. |
| 262 | Stanislawski (2023) | G6: Middleware integration and centralized LIMS | Designed and implemented a custom laboratory information management system (LIMS) and health information system (HIS) to manage samples and data. |
| 263 | Villanueva (2019) | G6: Middleware integration and centralized LIMS | To address the Zika data bottleneck, the LRN-B program office is implementing reporting procedures that require less lab staff time. |
| 264 | Williams (2014) | G6: Middleware integration and centralized LIMS | Have backup LIS and HIS systems, or well-planned manual procedures (e.g., logbooks), for when the primary system fails. |
| 265 | Yu (2010) | G6: Middleware integration and centralized LIMS | Deploy mobile satellite systems (e.g., MOBSAT) or wireless mesh networks (e.g., WIISARD) to rapidly restore connectivity. |
| 266 | Heller (2002) | G7: Secure, access-controlled digital networks | Regular third-party system vulnerability assessments and penetration testing are conducted. |
| 267 | Kennedy (2016) | G7: Secure, access-controlled digital networks | Secure, encrypted communication channels are established as the sole method for transmitting results. |
| 268 | Sandlin (2009) | G7: Secure, access-controlled digital networks | Mandatory, recurring cybersecurity training for all laboratory staff is implemented. |
| 269 | Sandlin (2009) | G7: Secure, access-controlled digital networks | Multi-factor authentication is implemented for all systems containing sensitive patient or laboratory data. |
| 270 | Sandlin (2009) | G7: Secure, access-controlled digital networks | Site-to-site Virtual Private Networks (VPNs) are used for secure data transfer between network laboratories. |
| 271 | Toney (2021) | G7: Secure, access-controlled digital networks | A formal incident response plan for cyber attacks is developed and tested. |
| 272 | Williams (2014) | G7: Secure, access-controlled digital networks | Always prepare for communication failure by having redundant systems and processes (e.g., amateur radio, satellite phones). |
| 273 | Williams (2014) | G7: Secure, access-controlled digital networks | Have backup LIS and HIS systems, or well-planned manual procedures (e.g., logbooks), for when the primary system fails. |
| 274 | Yu (2010) | G7: Secure, access-controlled digital networks | Disaster planning needs to account for the deployment of secure wireless networks (e.g., using WPA/WPA2 encryption). |
